# Neurological Immune Related Adverse Events Associated with Nivolumab, Ipilimumab, and Pembrolizumab Therapy—Review of the Literature and Future Outlook

**DOI:** 10.3390/jcm8111777

**Published:** 2019-10-24

**Authors:** Nora Möhn, Gernot Beutel, Ralf Gutzmer, Philipp Ivanyi, Imke Satzger, Thomas Skripuletz

**Affiliations:** 1 Department of Neurology, Hannover Medical School, Hannover 30625, Germany; moehn.nora@mh-hannover.de; 2 Center for Immuno-Oncology (IOZ) Hannover Medical School, Hannover 30625, Germany; beutel.gernot@mh-hannover.de (G.B.); gutzmer.ralf@mh-hannover.de (R.G.); ivanyi.philipp@mh-hannover.de (P.I.);; 3 Department of Hematology, Hemostasis, Oncology and Stem Cell Transplantation, Hannover Medical School, Hannover 30625, Germany; 4 Skin Cancer Center Hannover, Department of Dermatology and Allergy, Hannover Medical School, Hannover 30625, German

**Keywords:** immune checkpoint inhibitor therapy, nivolumab, pembrolizumab, ipilimumab, immune-related adverse events, encephalitis, myositis, Guillain-Barré syndrome, myasthenia gravis

## Abstract

Immune checkpoint inhibitor (ICI) therapy has revolutionized the management of various cancers with previously poor prognosis. Despite its great efficacy, the therapy is associated with a wide spectrum of immune-related adverse events (irAE) including neurological symptoms which can affect all parts of the central and peripheral nervous system. Even though these events are rare, they are of high relevance as the rate of residual symptoms or even fatal outcomes is remarkable. To provide a detailed overview of neurological adverse events associated with immune checkpoint-inhibitor therapy we conducted a literature search. While focusing on ipilimumab, nivolumab, and pembrolizumab therapy, all available case reports as well as larger case series and clinical trials have been considered. Eighty-two case reports about checkpoint-inhibitor therapy induced symptoms of the peripheral nervous system have been published, while only 43 case reports addressed central nervous system abnormalities. The frequency of immune checkpoint-inhibitor therapy inducing neurological adverse events is about 1% in larger studies. Especially neuromuscular adverse events exhibit distinct clinical and diagnostic characteristics. Additionally, several affected patients presented with overlap-syndromes, which means that symptoms and diagnostic findings indicating myositis, myasthenia gravis, and neuropathy were present in one individual patient at the same time. Thus, neurological and particularly neuromuscular adverse events of immune checkpoint-inhibitor therapy may constitute a new disease entity.

## 1. Introduction

The idea of targeting immune cells instead of cancer cells has led to a paradigm shift in oncology. Through antibody-mediated inhibitory interaction at certain immune checkpoint receptors, especially cytotoxic T-lymphocyte-associated antigen 4 (CTLA-4), programmed cell death protein (PD-1) and its ligand (PD-L1) anti-tumor immune responses are enhanced. Particularly T-cell mediated immunity is of major importance [1], but also antibody-or rather B-cell-mediated processes might be relevant [2]. This new therapeutic strategy has improved the prognosis of previously fatal diseases like metastatic melanoma [3]. To date, the CTLA-4 antibody ipilimumab, the anti-PD-1 antibodies nivolumab, pembrolizumab, cemiplimab, and the anti-PD ligand-1 antibodies atezolizumab, avelumab, and durvalumab have been approved for a variety of nine tumor entities. Despite the exceptional achievements in cancer therapy, impairing endogenous immunologic tolerance mechanisms can result in exaggerated uncontrolled immune response and development of autoimmune manifestations. In general, adverse events due to immune checkpoint blockade are frequent and will occur in up to 90% of anti-CTLA-4-antibody treated patients [3] and in about 70% of patients under treatment with anti-PD-1/anti-PD-L1 antibodies [4,5]. A recent meta-analysis including 125 clinical trials of PD-1 or PD-L1 inhibitors with 20,128 patients supported these findings. Within all included studies a total of 66.0% developed at least one adverse event of any grade, and 14.0% at least one grade 3 or higher adverse event [6]. Immune-related adverse events (irAEs) caused by reactivation of cellular immunity are of high relevance as they may affect every organ. Besides endocrine dysfunction the skin and the digestive tract are the most affected organs regarding immune checkpoint-inhibitor (ICI) treatment associated irAEs [7,8]. The meta-analysis by Wang and colleagues revealed an incidence of 6.07% for hypothyroidism, 9.47% for diarrhea, and 3.26% for vitiligo [6]. In patients suffering from metastatic melanoma, dermatologic irAEs are the most frequent ones [9,10]. Neurological adverse events (nAEs) are less frequent, whereby a high number of unreported cases has to be assumed as symptoms can be non-specific and difficult to interpret. When occurring, neurological irAEs can be fatal and affected patients often suffer from sequelae despite adequate therapy. Here, we aim to give an overview on published case reports and studies addressing nAEs due to ICI treatment, especially in treatment with nivolumab, ipilimumab, and pembrolizumab.

The occurrence of autoimmune adverse events due to immune checkpoint blockade was first described in animal models. CTLA-4 deleted mice exhibited symptoms of myocarditis or pancreatitis while mice lacking PD-1 developed arthritis, glomerulonephritis, or dilated cardiomyopathy [11,12,13]. T-cell dysregulation was held accountable for the development of these events. In humans irAEs were initially described in clinical trials of all CTLA-4 and PD-1 targeting therapies [5,9,10,14,15,16]. In addition, it is known that polymorphisms of PD-1 and CTLA-4 are associated with various autoimmune diseases such as thyroiditis [17], diabetes mellitus [17,18], rheumatoid arthritis [19,20], and even myasthenia gravis [21]. Disease susceptibility, for example in thyroiditis, is attributed to a non-coding region of the CTLA4 gene and a variation in gene splicing [17]. Whether a higher susceptibility to irAEs is caused by similar genetic alterations or whether the pathogenesis of irAEs is more diffuse needs to be addressed in further studies. Comparing rates of irAEs across different clinical trials may prove to be difficult as varied classification criteria for adverse events are used and the terminology for irAEs may differ, as well as frequency-cut-off values for reporting of irAE vary highly across the trials. In addition, follow-up periods and treatment doses varied within and between different studies which could as well affect the irAE detection rate [22]. A systematic review and meta-analysis of 13 multicentre studies comparing anti-PD-1 or anti-PD-1 ligand monoclonal antibodies versus standard treatment was published in 2018 [23]. Three thousand eighty-three patients who received ICI treatment were included. The most common irAEs were hypothyroidism (5.6%), pneumonitis (2.2%), colitis (0.7%), hypophysitis (0.3%), and hepatitis (0.2%). Even though the risk to develop colitis, pneumonitis, and hepatitis was higher compared with standard treatment, the overall incidence for irAEs in this specific clinical trial was surprisingly low. More unspecific but also allegedly immune related symptoms like fatigue, diarrhoea, and rash occurred more often (32%, 19%, and 10%, respectively). In general, mild symptoms (grade 1 and 2) are far more frequent and mainly affect the skin and the gut [24]. They usually resolve with systemic corticosteroid treatment. In the initial clinical trials, severe events were seen only incidentally [5,9,10,14,15,16]. IrAEs mostly occur within 3–6 months after initiation of ICI therapy [4,25], but delayed effects up to one year after start of anti-PD-1 treatment have been observed [26]. In anti-CTLA-4 treatment the risk of irAEs seems to be dose-dependent [10,27], while anti-PD-1 therapies apparently boast no cumulative toxicities [4]. Generally accepted, irAE rates are higher in patients treated with CTLA-4 antibodies than with PD-1 blockade [28]. The following sections will demonstrate that this does not apply for nAEs.

## 2. Search Strategy

The literature search was conducted on the PubMed database. Search terms: Neurological adverse events checkpoint inhibitor, checkpoint inhibitor neurology, neurological toxicities checkpoint, neurological toxicities PD-1/CTLA-4, nivolumab neurological adverse events, ipilimumab neurological adverse events, PD-1 neurological adverse events, PD-1 myasthenia gravis, PD-1 GBS, PD-1 neuropathy, checkpoint inhibitor myasthenia gravis, checkpoint inhibitor GBS, checkpoint inhibitor neuropathy, checkpoint inhibitor myositis, “encephalitis nivolumab”, and “encephalitis pembrolizumab”, “encephalitis ipilimumab”, nivolumab.

## 3. Neurological irAEs (nAE)

In prospective clinical trials (phase I, II, and III) the incidence of any grade nAEs including unspecific symptoms like headache and dizziness varied between 0% and 27% for both anti-CTLA-4- and anti-PD-1 treatment [7] with a median nAE-incidence of 3.8% in ipilimumab and tremelimumab treated patients and of 6.1% in patients who received anti-PD1 therapy. This result is in contrast to the fact that irAEs in general are more common during anti-CTLA-4 therapies [28]. Clearly higher nAE incidence rates are observed for a combination therapy with anti-CTLA-4 and anti-PD1 treatment [7]. However, incidence of high grade (grade 3–4) nAEs in clinical trials was below 1% even in patients having received combination therapy [3,7]. As large, prospective investigations are completely missing, reported incidences of nAEs vary significantly among the publications and case selection as well as reporting bias must be considered. For example, there are pooled analyses reporting any grade nAE overall incidences of only 0.1% [29] which was considered to be an underestimation, though [30]. In contrast to this, Spain and colleagues presented their results of a five-year single center experience at Royal Mardsen hospital in London where 413 melanoma patients received ICI-therapy.

Overall incidence of neurotoxicity in this study amounted to 2.4% (10 patients) while severe nAEs (grade 3 and 4) occurred in six cases (1.45%). Nivolumab-treated patients were affected more often compared with patients who received ipilimumab or pembrolizumab (7% vs. 1% and 2%) and combination therapy harboured the highest risk for development of nAEs (14%) [31]. A 2016 published retrospective series of 496 melanoma patients who were treated in 15 centers either with nivolumab or pembrolizumab serves as an example of reporting bias. It reported only 16 cases of neurological side effects, but symptoms of the musculoskeletal system (21 cases) and eyes (eight cases) were not considered as nAEs; therefore, the actual incidence was most probably higher [32]. This highlights the challenges of measuring nAE incidence in large clinical studies. 

ICI-treated patients with pre-existing neurological autoimmune disorders represent a special group of persons. Garcia and colleagues identified 14 cases of multiple sclerosis when analysing the United States Food and Drug Administration Adverse Event Reporting System (FAERS) database including eight patients with confirmed history of multiple sclerosis [33]. All patients presented with rapid neurological disease progression and required medical intervention. Disease presentation was particularly severe in three cases and two patients even died from their multiple sclerosis relapse [33]. Yuen and colleagues published a case of a patient who developed a severe and fatal relapse of postvaccination Guillain-Barré syndrome after treatment with nivolumab [34]. As much is still unknown regarding risk factors for developing nAEs a history of autoimmune disorders as a contraindication for ICI-therapy has to be carefully considered.

The following sections describe the characteristics of nAEs in detail. For this purpose, we provide an overview of published case reports regarding nAEs of the central and peripheral nervous system published up to June 2019. 

## 4. Central Nervous System Manifestations

Compared with symptoms of the peripheral nervous system (PNS), central nervous system (CNS) manifestations of nAEs are even rarer. They are mostly described in small case studies and especially in case reports (Table S1). As every neurologist knows, symptoms of the CNS such as headache, fatigue, dizziness, or confusion may be very unspecific. Therefore, it can be assumed that many cases remain unreported. In 2017 Larkin and colleagues published a case series of six cases of encephalitis that occurred within the pivotal studies of nivolumab and ipilimumab [35]. One 55-year old female patient was described as a case report by Williams and colleagues as well (see Table S1). Patients in these series had a mean age of 61.3 years (range: 53–83 years). Two patients were male and four were female. In three cases, the patients had received a combination therapy of ipilimumab and nivolumab, while two patients got nivolumab monotherapy and one man had obtained sequential nivolumab and ipilimumab application followed by nivolumab monotherapy. The mean time to onset of symptoms from study-drug initiation amounted to 94.8 days (range: 18–297 days). Most patients exhibited features of an altered mental state such as confusion, aphasia, disorientation, and agitation [35]. In four patients, the results of cerebrospinal fluid (CSF) analysis were documented and interestingly three of four patients showed elevated white blood cell count. In contrast to this quite homogenous group of patients, CNS manifestations of patients reported by Kao and colleagues in 2017 were highly variable. In their single-centre, retrospective cohort study 10 out of 347 patients (3%) treated with pembrolizumab or nivolumab developed neurological symptoms. In three cases, CNS manifestation was described [36]. Patients presented with cerebellar ataxia and dysarthria, bilateral internuclear ophthalmoplegia, and acute onset of severe headache. In all cases, radiological and laboratory diagnostics (including CSF analysis) were unremarkable. Symptoms improved after low dose steroid therapy. In one case, discontinuation of anti-PD-1 treatment was sufficient and no further immunosuppressive therapy was necessary [36]. Galmiche and colleagues conducted a retrospective observational study of 209 melanoma patients treated with ICI regarding encephalitis. Five of 209 patients (2.4%) presented with ICI-induced encephalitis with a median interval between ICI initiation and symptoms onset of 42 days, whereby symptoms occurred later in patients treated with pembrolizumab [37]. Remarkably, only three patients displayed full resolution of symptoms [37].

**Figure 1:**
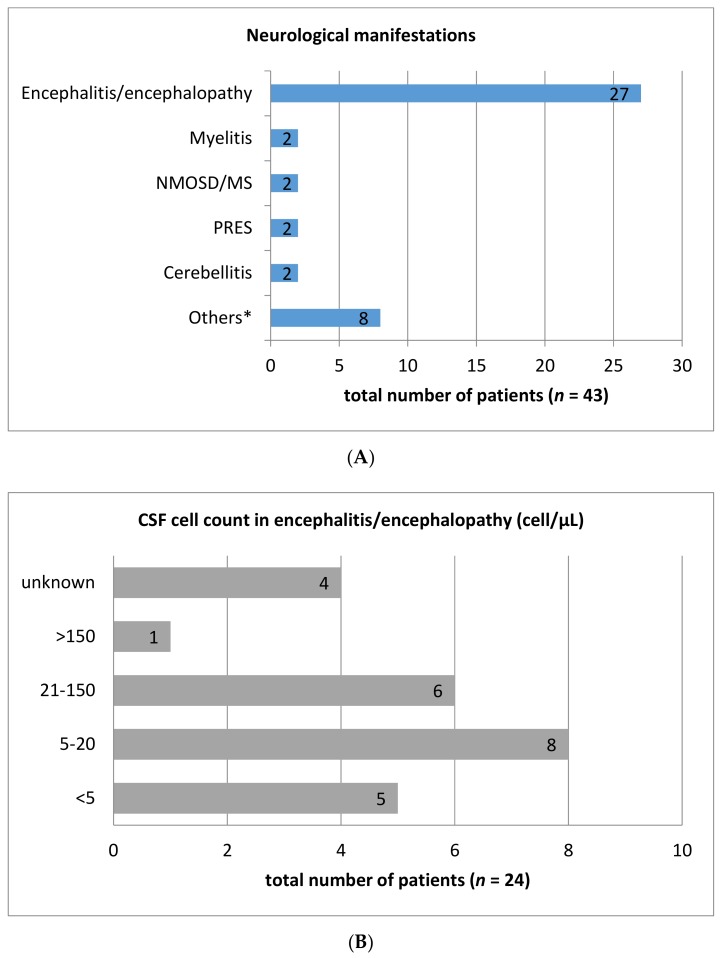

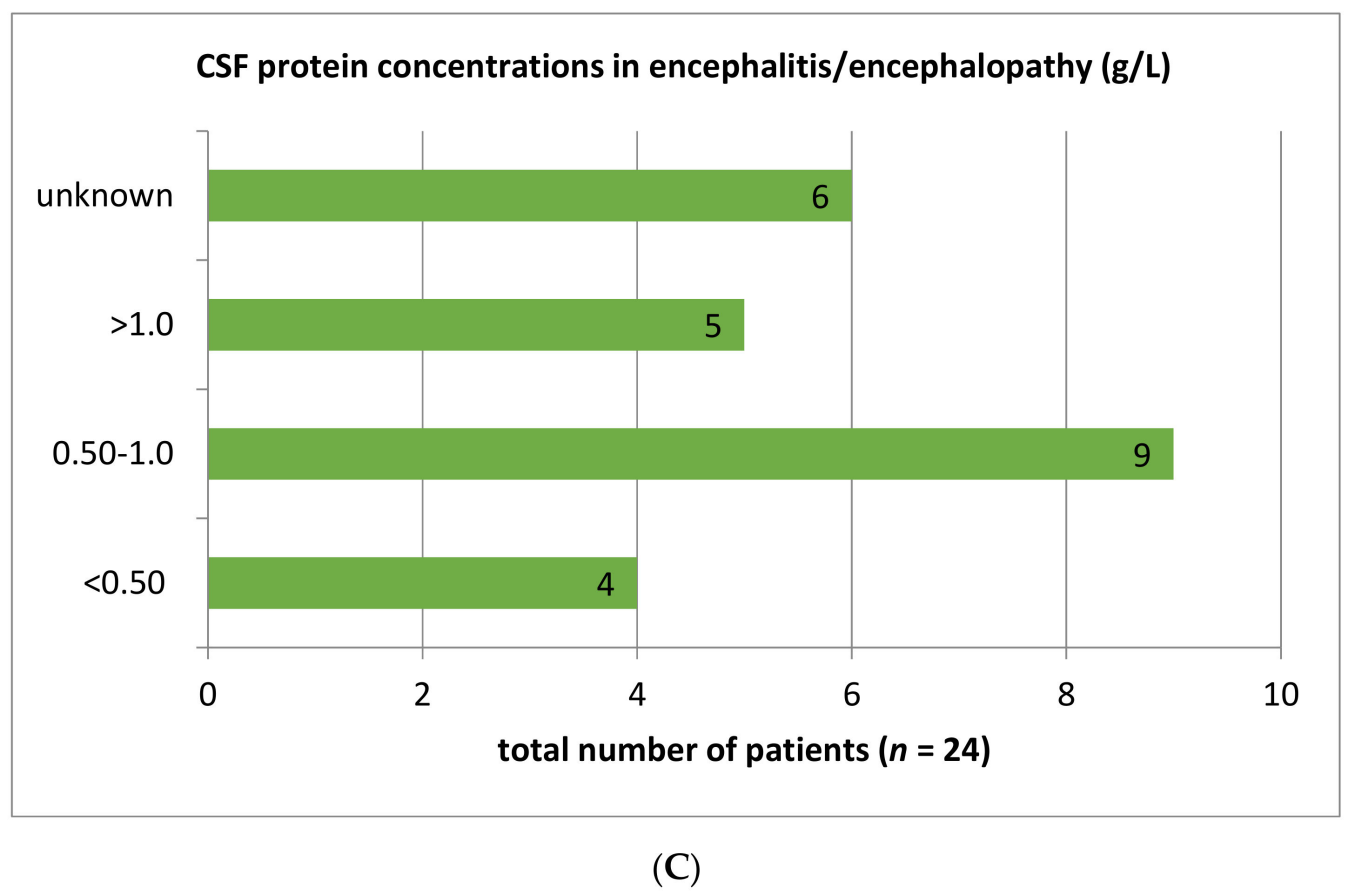
Number of different central nervous system manifestations in a total of 43 case reports of immune checkpoint-inhibitor (ICI)-mediated neurological adverse events (**A**). Measured cerebrospinal fluid (CSF) cell count (**B**) and protein concentrations (**C**) in the most common entity, encephalitis/encephalopathy. CSF cell count was analysed in 24 of 27 case reports with encephalitis/encephalopathy. CSF: cerebrospinal fluid; MS: Multiple sclerosis; NMOSD: Neuromyelitis optica spectrum disorder; PRES: Posterior reversible encephalopathy syndrome. Others*: Meningitis (2 cases), neurosarcoidosis (1 case), meningo-radiculo-neuritis (1 case), cerebral vasculitis (1 case), PML (1 case), central facial palsy (1 case), and brain lesion mimicking brain abscess (1 case).

Besides these case series, CNS complications of ICI therapy have been presented in several case reports. Table S1 illustrates the published case reports up to 06/2019 regarding CNS symptoms during treatment with ipilimumab, nivolumab, and pembrolizumab. A total of 43 cases have been reported, among them 27 patients with encephalitis, two cases of cerebellitis, myelitis, and posterior reversible encephalopathy syndrome (PRES), respectively. Single case reports for meningitis, neurosarcoidosis, multiple sclerosis, neuromyelitis optica spectrum disorder (NMOSD), and meningo-radiculo-neuritis have been described as well (Figure 1A). Interestingly, gender distribution is male dominated as 31 of 43 cases (72%) are men. The mean age of patients in published cases amounted to 55.6 years (range: 26–78 years). While the most frequent indication for ICI-therapy was metastatic melanoma (44.2%), 17 patients (39.5%) were treated with nivolumab and 11 patients (25.6%) received ipilimumab monotherapy. Six patients (14%) were treated with pembrolizumab monotherapy. Combined therapy with nivolumab and ipilimumab or pembrolizumab and ipilimumab was administered in six (13.9%) and two (4.7%) cases, respectively. One patient (2%) received combination therapy with ipilimumab and lambrolizumab. Other underlying diseases were lung carcinoma (27.9%), lymphoma (11.6%), and renal cell carcinoma (7.0%), as well as single cases suffering from prostate cancer, ovarian cancer, laryngeal carcinoma, or pleura mesothelioma. Regarding the diagnostic methods, it should be noted that lumbar puncture was conducted in 34 patients (79%). In 60.5% of cases (*n* = 26) CSF analysis revealed elevated cell count ranging from six to 1195 cells/µL. The majority of patients exhibited a cell count between six and 150 cells (84.5%; *n* = 22) (Figure 1B). 22 patients (65%) exhibited elevated CSF protein concentration (range: 0.56 g/L–5 g/L) (Figure 1C). Most (88.3%; *n* = 38) nAEs of the CNS were treated with steroids in various dosages. Most patients received high dose (>1 mg/kg bodyweight) intravenous methylprednisolone. In nine (20.9%) and six (14.0%) cases intravenous immunoglobulins and plasmapheresis were applied in addition, respectively. In five cases no treatment was initiated. Following immunosuppressive therapy 16 patients (37.2%) achieved complete remission or major improvement of immune-related symptoms. Partial improvement was attained in 41.9% (*n* = 18). Unfortunately, most case reports did not quantify the residual symptoms, so that an accurate assessment of disability was not feasible. Seven patients had no amelioration of symptoms or died despite initiation of immunosuppressive treatment. Two patients were lost to follow-up.

## 5. Peripheral Nervous System Complications

Neuromuscular complications of ICI-therapy are the most frequent neurological manifestations with myasthenia gravis being characterized as the most common PD-1 inhibitor-associated neuromuscular complication [38,39]. Patients with ICI-induced myasthenia gravis can present with positive as well as negative acetylcholine receptor (AChR)-antibodies. However, about 25% of reported patients had been diagnosed with myasthenia gravis before and suffered a relapse following ICI-administration [39]. ICI-therapy induced Guillain-Barré syndrome is another severe irAE of the peripheral nervous system. Reflecting upon the characteristics of published case reports (Table S2), it becomes obvious that clinical presentation, course, and electrophysiological findings resemble those of not-ICI-related Guillain-Barré syndrome [40,41,42,43,44]. However, relatively frequently patients with ICI-induced Guillain-Barré syndrome exhibit an elevated CSF cell count [39,45], while classical Guillain-Barré syndrome patients usually do not show significant CSF pleocytosis [46]. Of course, other causal entities such as viral infections with Campylobacter jejuni, Cytomegalovirus (CMV), Epstein-Barr virus (EBV), HIV, and Zika virus which can be accompanied by GBS-like symptoms and CSF pleocytosis need to be excluded. Compared with Guillain-Barré syndrome, chronic inflammatory demyelinating polyneuropathy is much less commonly reported. Up to now, three cases of melanoma patients with ICI-related chronic inflammatory demyelinating polyneuropathy have been published (Table S2) [30,42,47]. In two further cases of melanoma, patients under ipilimumab-therapy developed symmetric painful paraesthesia of the feet, gait instability, and weakness of the lower limbs, without being defined as chronic inflammatory demyelinating polyneuropathy [48]. Two additional case reports mentioned polyneuropathic symptoms that manifested as limb weakness and sensory deficits after nivolumab and pembrolizumab treatment, respectively [49,50]. Interestingly, immunosuppressive therapy in patients with ICI-related polyneuropathy was highly variable. All patients were treated with steroids (i.v. or oral), while two got additional therapy with intravenous immunoglobulins [47,48]. In one patient plasma exchange was performed with limited success [42] and two affected persons obtained other immunosuppressive drugs like infliximab, tacrolimus, or mycophenolate mofetil [48].

Estimated frequency of muscular symptoms in two large series with 347 and 654 PD-1 treated patients amounted to 0.6% and 0.8%, respectively [36,51]. Kao and colleagues identified 10 patients out of 347 (2.9%) with neurological complications related to the treatment with nivolumab or pembrolizumab. The patients’ median age was 71 years (age range, 31–78 years). As expected, malignant melanoma as the most frequent indication for ICI-therapy was the most common underlying disease (*n* = 5), followed by lung adenocarcinoma (*n* = 2), peritoneal mesothelioma (*n* = 1), esophageal adenocarcinoma (*n* = 1), and leiomyosarcoma (*n* = 1) with all patients suffering from stage IV metastatic disease. Among 10 patients with PD-1-associated nAEs, neuromuscular disorders were the most common neurological complications and included myopathy (*n* = 2) and neuropathy (*n* = 4) [36].

Among the 654 PD-1 treated (pembrolizumab = 389; nivolumab = 264; both = 1) cancer patients described by Liewluck and colleagues, the authors identified five patients (pembrolizumab = 5) with biopsy-proven myopathies (two necrotizing myopathy, one early dermatomyositis, and two nonspecific myopathy). All five patients presented with proximal or axial weakness which occurred after a median of two treatment cycles (range, 1–4) [51]. Four patients exhibited additional bulbar or extraocular weakness. Pembrolizumab was discontinued in all patients and they all received additional immunosuppressive therapy. However, in two patients with necrotizing myopathy, the course of disease was fatal which could indicate that necrotizing myopathy patients may require more aggressive immunotherapy. Overall, proximal or generalized limb weakness, ptosis or diplopia, and myalgia are the most frequently reported symptoms in patients with ICI-induced muscular disorders (Table S2). Dyspnoea, dysphagia, or dysarthria occur less frequently. The high frequency of ocular involvement is typical for ICI-associated myopathies [51] and may result in the diagnosis of concomitant myasthenia gravis in several cases. Distinguishing between ocular symptoms as a direct effect in the sense of orbital myositis or as an expression of possible concomitant myasthenia gravis is difficult [52,53]. Muscle biopsy might be a helpful diagnostic tool to differentiate patients with necrotizing myopathy from patients with myasthenia gravis. Besides myasthenia gravis, myositis can be accompanied by myocarditis [54,55,56]. Johnson and colleagues published a series of 101 PD-1-treated patients who suffered from myocarditis. Interestingly, in 25% of the cases myocarditis occurred in combination with myositis and 10 patients had concomitant myasthenia gravis [57]. Creatine kinase levels can be within normal range; however, the median CK level in Johnsons cohort amounted to 2566 U/L and maximum value was 30,980 U/L [57]. In most patients with PD-1 associated myopathies, histopathological findings were similar to muscle biopsy results of patients with other myopathies. In PD-1-induced necrotizing autoimmune myopathy biopsies showed a multifocal pattern of necrotic fibres compared to a more generalized pattern in patients with sporadic necrotizing autoimmune myopathy [51,58]. Knauss and colleagues were able to demonstrate that in immune related myositis, T cell invaded the skeletal muscle and myofibers concomitantly upregulated ligands that stimulated PD-1. The invading T cells show an exhausted phenotype with high expression of PD-1, LAG-3, and TIM-3 [59].

Recently, Moreira and colleagues published results from their side-effect-registry which included irAE-reports from 10 skin cancer centres in Germany and Switzerland with an observation period of 5.5 years. A total of 38 melanoma patients had reported neuromuscular side-effects associated with ICI-therapy during this period [55]. This was the first German-speaking area scientific work which specifically focused on neuromuscular side effects of ICI therapy. The median latency between first ICI infusion and onset of symptoms was 19 weeks with a highly variable range of one to 115 weeks. In contrast to previous reports [38], the majority of patients presented with symptoms of myositis with and without myocardial involvement. All but one patient was diagnosed with myasthenia gravis [55]. Interestingly, only half of the cases completely resolved despite immunosuppressive therapy with steroids or intravenous immunoglobulin. Apart from two deaths, the patients suffered from sequelae or had ongoing symptoms; a fact which emphasizes the importance of neuromuscular side effects in the context of ICI-treatment. 

One fact applies to all nAEs of the peripheral nervous system: the underlying mechanisms and their pathogenesis have not been fully elucidated yet. It can be assumed that activation of cytotoxic T lymphocytes and simultaneous reduced suppression of autoantibody-producing B lymphocytes through PD-1- or CTLA-4-inhibition may lead to formation of autoantibodies against neuronal structures [39]. In the case of ICI-related Guillain-Barré syndrome (and chronic inflammatory demyelinating polyneuropathy), it has been postulated that especially melanoma patients are affected as melanocytes and Schwann-cells express some similar epitopes [30,44,60]. However, as by far not all patients with ICI-associated nAEs exhibit autoantibodies, further unknown mechanisms have to be presumed.

**Figure 2:**
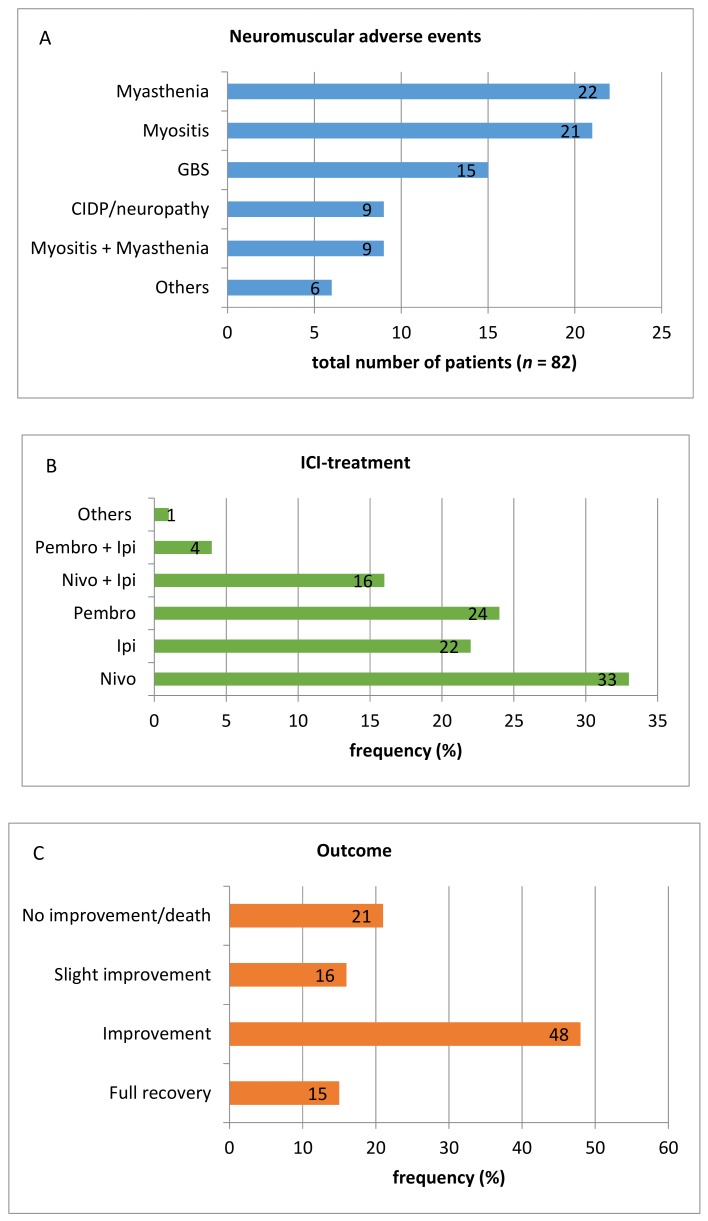
Number of different neuromuscular adverse events (**A**), frequency distribution of underlying ICI-therapy (**B**), and outcome after neuromuscular adverse events (**C**) in analysed case reports of ICI-mediated neurological adverse events. CIDP: Chronic inflammatory demyelinating polyneuropathy; GBS: Guillain-Barré syndrome; ICI: Immune-checkpoint inhibitor; Ipi: Ipilimumab; Nivo: Nivolumab; Pembro: Pembrolizumab.

Between 2011 and 2019 several case reports regarding neuromuscular adverse events of ICI therapy have been published. Table S2 contains information about 82 case reports providing a representative overview of the high variability of symptoms that can occur during treatment with ipilimumab (*n* = 18), nivolumab (*n* = 27), pembrolizumab (*n* = 20), or a combination of either ipilimumab and nivolumab (*n* = 13) or ipilimumab and pembrolizumab (*n* = 3). One single patient who developed myopathy involving the extraocular muscles was treated with a combination of tremelimumab and durvalumab (Figure 2B). As nivolumab and ipilimumab are available for much longer than pembrolizumab, the frequency of pembrolizumab-induced side effects appears unusually high. The fact that a higher number of nivolumab-treated patients developed neurotoxicity is consistent with findings of Cuzzubbo and colleagues who reported a higher incidence of nAEs during anti-PD1 therapy. Considering all published cases, myasthenia gravis and myositis are the most common immune-related neuromuscular adverse events during ICI-therapy amounting to 26.8% (*n* = 22) and 25.6% (*n* = 21), respectively. With a frequency of 18.3% (*n* = 15) Guillain-Barré syndrome proved to be the third most common neuromuscular adverse events. A combination of symptoms indicating both myasthenia gravis and myositis occurred in nine patients (11%). Nine patients were affected by chronic neuropathies such as chronic inflammatory demyelinating polyneuropathy (CIDP). Another six singular case reports described less common symptoms like Bell’s palsy or bilateral phrenic nerve neuropathy (Figure 2A). The patients’ mean age was 65.7 years (range: 34–88 years) and men were slightly more affected than women (57% vs. 43%), which primarily seems to explained by the fact that in general more men are treated with ICI-therapy. The gender distribution in larger clinical studies amounts to around 60%:40% (m:f) [3]. Metastatic melanoma is by far the most common underlying disease (*n* = 59). Other underlying diseases are lung carcinoma including non-small cell lung carcinoma (NSCLC) (*n* = 12), bladder and renal cell carcinoma (*n* = 4), and others (*n* = 7), for example, Hodgkin’s lymphoma, thymic cancer, or uterine carcinosarcoma.

Diagnostic procedures (for example, CSF analysis, MR imaging, neurography) considerably vary among the different case reports. In 30 patients (36.6%) lumbar puncture has been performed. Of those, 19 patients exhibited elevated CSF-protein and 10 patients had an elevated CSF cell count. Almost all affected persons received oral or intravenous steroid therapy (*n* = 70; 85.4%). Intravenous immunoglobulins have been administered in 39 patients (47.6%) and plasma exchange was performed in 28% of cases (*n* = 23). In individual cases, immunosuppressive treatment options were combined with methylprednisolone and intravenous immunoglobulins, or methylprednisolone and plasma exchange as the most common combinations. Besides those options mentioned, other immunosuppressive drugs like infliximab, tacrolimus, mycophenolate, or rituximab have been applied. By far not all cases of immune-related neuromuscular adverse events were successfully treated. In 21.3 % of analysed case reports (*n* = 17), patients showed no improvement or even died. Only 12 patients (14.6%) fully recovered (Figure 2C). Most of these results are in line with a systematic review regarding neuromuscular adverse events associated with anti-PD-1 monoclonal antibodies conducted by Johansen and colleagues in 2019 [61].

## 6. Overlap-Syndromes: A New Disease Entity?

One of the most interesting findings when analyzing ICI-induced nAEs is the joint appearance of myasthenic, myositic, and neuropathic symptoms [38,45,55,61]. Patients may exhibit specific AChR-antibodies and simultaneously show symptoms of demyelinating neuropathies and acute muscle inflammation consistent with myositis [45]. Although the coexistence of inflammatory myopathy and myasthenia gravis was described before ICI-therapy cleaved its way, the frequency of such simultaneous occurrences was much lower [62,63]. Compared to a maximum frequency of myocarditis in 0.35% of PD-1-naïve myasthenia gravis patients, up to 25% of PD-1-treated patients may show a simultaneous appearance of myositis and myasthenia gravis [54]. Overall, it appears that immune-related neuromuscular adverse events have specific characteristics. As an example, unlike other forms of myositis, immune-related myositis often manifests with oculomotor weakness [56], which may further complicate the differentiation between myositis and myasthenia gravis.

A further important point to note is that a high number of patients with neuromuscular side effects show signs of cardiac involvement. Within the German and Swiss irAE side-effect-registry, 32% of all myositis cases were accompanied by myocarditis [22,55]. Immune-related myositis can be a fatal adverse event of ICI-therapy, especially when it comes along with cardiac involvement [64]. Therefore, an expeditious and profound diagnosis and consecutive initiation of immunosuppressive therapy is of major importance. In inconclusive cases, peripheral muscle biopsy proved to be an appropriate tool to confirm the diagnosis of ICI-induced myositis. Findings of histopathologic analyses may vary, though. Within the cohort described by Moreira and colleagues muscle biopsies showed infiltrates dominated by CD4+ and CD20+ lymphocytes as well as necrotising myopathic changes [55]. Necrotic fibers were also observed by Johansen et al., but biopsies of their patients exhibited a dominance of CD8+ lymphocytes [61]. Touat and colleagues detected densely clustered myophagocytoses with numerous macrophages in endomysium and perimysium as well as inflammatory infiltrates consisting mainly of CD68+ and CD8+ cells [56]. In conclusion, neuromuscular adverse events associated with ICI-therapy harbor unique clinical and diagnostic features compared with sporadic myositis, myasthenia gravis, or inflammatory neuropathy. In particular, the frequent coincidence of those diseases in ICI-treated patients is remarkable. It could be concluded that neuromuscular adverse events induced by ICI-therapy may represent a new disease entity with implications on treatment and prognosis.

## 7. Recommendations for Management of Neurological Adverse Events

In general, we see an obvious need for focused, structured, and standardized investigations regarding potentially fatal adverse events of ICI-therapy. However, it is difficult to derive specific recommendations for actions from the presented case reports. Checkpoint inhibitor therapy should be discontinued if higher-grade adverse events are suspected. It has been previously described that administration of steroids (dosage 0.5–2 mg/kg prednisolone or equivalent) can improve the course of higher-grade neurological and non-neurological adverse events [3]. Based on our experiences and on the results of the presented cases we can state that a treatment with prednisolone is often not sufficient to control neurological deficits. These observations are in line with previous reports that intravenous immunoglobulins or plasmapheresis should be considered in case of steroid-insufficiency [3,65]. We therefore suggest a low threshold for the indication of intravenous immunoglobulins in patients with nAEs, especially in patients with symptoms indicating a neuropathy-myositis-myasthenia-overlap syndrome. In individual and particularly serious cases, plasma exchange could also be an option.

## 8. Conclusions

Neurological adverse events are infrequent but highly relevant complications of ICI-therapy as they can lead to long-term disability or death. Compared with central nervous system disorders, symptoms of the peripheral nervous system have been described more often and in more detail. Exhibiting unique characteristics and especially overlapping symptoms, immune-related neuromuscular adverse events seem to define a new disease entity. Besides medical imaging or electroneurography, diagnostic work-up should include CSF-analysis since several patients with nAEs exhibit unexpectedly high CSF cell counts. So far, little is known about the pathogenesis and potential risk factors of nAEs. Most of the reported events seem to respond well to immunosuppressive treatment with steroids, but there is no structured guideline for steroid-refractory cases. This review gives an overview of several case reports and larger clinical studies that reported nAEs due to ICI-therapy. However, the selection bias during literature search leads to unavoidable biases that have to be considered when interpreting the results of this article and drawing conclusions from them. Future considerations have to address the following questions: What are the exact mechanisms behind nAEs in ICI-treated patients? Are there pre-treatment risk factors that help to estimate the risk of developing nAEs for every individual patient? Does the occurrence of irAEs correlate with the antitumor effect of ICI-therapy? What is the right immunosuppressive treatment-strategy in the case of nAE development and do we need an individual treatment approach for every patient? However, until we know the answers to these questions, interdisciplinary management of oncologist and neurologist is mandatory in neurological irAEs.

## Supplementary Materials

The following are available online at https://www.mdpi.com/2077-0383/8/11/1777/s1, Table S1: Overview of published case-reports regarding central nervous system manifestations as neurological adverse events of ICI-therapy, Table S2: Overview of published case-reports regarding peripheral nervous system manifestations as neurological adverse events of ICI-therapy.
