# Supplementary material for: Neurological Immune Related Adverse Events Associated with Nivolumab, Ipilimumab, and Pembrolizumab Therapy—Review of the Literature and Future Outlook
Source: J Clin Med. 2019 Oct 24;8(11):1777. doi: 10.3390/jcm8111777 (PMC6912719; doi:10.3390/jcm8111777)
Supplement: Supplementary file 1 [file jcm-08-01777-s001.pdf]

Journal of Clinical Medicine

MDPI

Table S1. Overview of published case-reports regarding central nervous system manifestations as neurological adverse events of ICI-therapy. Besides patients' clinical data certain diagnostic findings as well as immunosuppressive.

author	year	patient age	patient sex	underlying disease	ICI therapy	neurological symptoms	latency ICI-start/onset irAE	CSF findings	brain MRI	antibody findings	immunosppr. therapy	outcome	others
**Bossart et al [1]**	2017	60	female	metastatic melanoma, cerebral metastases	ipilimumab + pembrolizumab	**encephalitis** (generalized weakness, tiredness)	12 weeks	n.d.	inconspicuous	n.d.	n.d.	death	autopsy: infiltrates of CD8+ lymphocytes esp. In brainstem
**Burke et al [2]**	2018	64	female	clera-cell ovarian cancer	nivolumab	**encephalitis** (fever, spasms, delirium)	17 weeks	n.d.	inconspicuous	GAD-antibody positive	methylpredn. i.v., plasmapheresis	major improvement	
**Maurice et al [3]**	2015	60	male	metastatic melanoma	ipilimumab + nivolumab	**encephalitis** (subacute confusion, nausea, vomiting, psychomotor slowing)	8 weeks	cc 0, protein 0.88 g/l, myelin-basic protein positive	tumefactive demyelination	n.d.	methylpredn. i.v., IVIG	improvement	CD4+ and CD8+ T-cell infiltration of spinal cord
**Richard et al [4]**	2017	74	male	NSCLC	nivolumab	**encephalitis** (dysathria, weakness of lower limbs)	1 week	inconspicuous	n.d.	negative	methylpredn. i.v.	improvement	
**Schneider et al [5]**	2017	78	male	squamous cell carcinoma lung	nivolumab	**encephalitis** (apathy, aphasia, myoclonuses)	28 weeks	cc 16, lactate 4.1 mol/l, protein 1.03 g/l	inconspicuous	negative	methylpredn. i.v.	major improvement	
**Williams et al [6]**	2016	55	female	metastatic melanoma, cerebral metastases	nivolumab + ipilimumab	**encephalitis** (memory loss, gait disturbance, fever, inappropriate laughing)	1 week	cc 8, glucose and protein normal	inconspicuous	NMDA-R-antibodies positive in CSF	methylpredn. i.v., IVIG	no improvement, after rituximab	
		65	male	SCLC	nivolumab + ipilimuma	**encephalitis** (short-term memory loss,	4 days	cc 18, protein 0.98 g/l	nonspecific T2 hyperinten	antiglial nuclear antibody	prednisone 60 mg/d	dramatic improvement	

				b	progressive gait disturbance)			se lesion right mesial ltemporal lobe	(SOX-1), serum			
**Kawamura et al [7]**	2016	54	female	adeno-carcinoma lung, cerebral metastases	nivolumab	**cerebellitis** (dizziness, nausea, nystagmus, cerebellar ataxia)	2 weeks	cc 10, protein 0.56 g/l	inconspicuous	negative	methylpredn. 1g/d, 3 days	major improvement	patient died due to pneumonia
**Narumi et al [8]**	2018	75	male	squamous cell carcinoma lung	nivolumab	**NMOSD** (acute bilateral paralysis, sensory loss below Th10)	8 weeks	cc 1195, protein 3.8 g/l	brain MRI: inconspicious; **spinal**: T2 hyperintense lesions C5-6, Th12-L1	AQP4, serum	methylpredn. i.v., plasmapheresis	minimal improvement	
**O'Kane et al [9]**	2014	58	male	metastatic melanoma	ipilimumab	**Myelitis** (weakness left leg, sensory deficite below Th10, constipation, micturition disturbance)	24 weeks	cc 16, protein 0.57 g/l	brain MRI: inconspicious; **spinal**: T2 hyperintense lesion Th7-L1	negative	methylpredn. i.v., IVIG 5 days	little improvement	colitis, day 16 after therapy start
**Tan et al [10]**	2018	65	male	metastatic melanoma	nivolumab + ipilimumab	**neurosarcoidosis** (transient aphasia, visual field defecits, mental status deficits)	52 weeks	cc 13, protein 0.75 g/l	leptomeningeal enhancement left occipital and parietal lobes	n.d.	methylpredn. i.v., dexamethasone 16 mg/d; infliximab 5mg/kg; MTX 12,5 mg/week	improvement	
**Salam et a [11]**	2016	64	male	metastatic melanoma	pembrolizumab	**limbic encephalitis** (progressive decline in memory MMSE 22/30)	52 weeks	cc 17, protein 0.53 g/l	bilateral symmetrical T2 hyperintens., hippocampi, anterior temporal lobe, insula atrophy	negative	methylpredn. i.v.	no improvement, no decline	
**Stein et al [12]**	2015	56	male	metastatic melanoma	ipilimumab	**meningoencephalitis** (diaphoresis,	15 weeks	cc 705 (96%	initially negative;		methylpredn. i.v.	complete recovery	

						fevers, declining mental status)		lymphos) protein 2.2 g/l	Repeat: abnormal diffuse dural thickening with enhancement		160 mg/d	
**Khoja et al [13]**	2016	51	female	metastatic melanoma	pembrolizumab	**acute encephalopathy** and eosinophilic fasciitis (headache, visual field floaters, movement difficulty; thickened waxy skin; confusion)	76 weeks	cc normal, protein 0.27 g/l	hyperintense white matter foci subcortical and periventricular, all with restricted diffusion	n.d.	methylpredn. i.v.	slight improvement
**Gettings et al [14]**	2015	56	male	metastatic melanoma	ipilimumab	**MS relapse** (new gait dysfunction, ataxia)	4 weeks	n.d.	new enhancing lesion centrum semiovale left; follow-up: two new lesions	n.d.	methylpredn. i.v. 500 mg 3 days; glatiramer acetate + steroids	
**LaPorte et al [15]**	2017	26	female	Hodgkin's Lymphoma	ipilimumab, pembrolizumab	**PRES** (grand mal seizure)	1.5 weeks	n.d.	FLAIR hyperintensity parasagittal, frontoparietal, temporal-occipital	n.d.	none	improvement
**Maur et al [16]**	2012	58	female	metastatic melanoma	ipilimumab	**PRES** (acute blindness, headache, generalized seizures)	2 weeks	n.d.	FLAIR hyperintens lesions posterior white-matter region: cerebellar,	n.d.	cortisone acetate (s. adrenohypophysitis)	complete recovery

									parieto-ocipital				
**Mandel et al [17]**	2014	66	male	metastatic melanoma	ipilimumab, lambrolizumab	ataxia, vertigo, numbness left arm; convulsive status	10-12 weeks	cc 6, protein 1.03 g/l	1. brain MRI: unremarkable (motion artifact); 2. brain MRI: FLAIR hyperintensities bilat. Claustrum + right frontal and left occipital lobes	n.d.	none (anticonvulsive therapy)	complete recovery	open biops, lymphocytic infiltratesy of right frontal lesion: diffuse microglial activation
**Bompaire et al [18]**	2012	56	male	metastatic melanoma	ipilimumab	**meningo-radiculo-neuritis** (vertigo, diziness, headache; rep. falls, dysarthria, dysaesthesia; severe gait ataxia, tetraplegia)	10 weeks	1. cc 135, protein 5 g/l; 2. cc 104, protein 4.45 g/l	brain MRI: inconspicuous; **spinal** MRI: global enhancement of nerve-roots	negative	oral prednisone 80 mg/d; methylpredn. i.v. 1g/d 3 days, IVIG 0,4g/kg/d 5 days + oral prednisone 1 mg/kg/d 4 months	almost complete recovery after 24 months	two conduction blocs (left perone, right cubital), acute deervation lower limbs
**Altman et al [19]**	2015	32	male	metastatic melanoma	ipilimumab	**bilateral facial palsy**	5 weeks	n.d.	n.d.	n.d.	oral prednisone 80- 100 mg/d	improvement	
**Carl et al [20]**	2015	64	male	prostate cancer	ipilimumab	hypophysitis 11/2011; **encephalopathy** 08/2012 + autoimmune thyroiditis: SREAT (adynamia, memory disturbances; myocloni,	52 weeks	cc normal, protein 0,85 g/l	mild microangiopathic changes	negative	oral prednisolone 50 mg/d; methylpredn. i.v. 1g/d 3 days	major improvement	

						seizures)						
**Conry et al [21]**	2015	41	male	metastatic melanoma	ipilimumab	**encephalopathy** (fever, headache, myalgia; expressive aphasia, gait ataxia), peripheral symptoms (sensory neuropathy, neurogenic bladder)	7.5 weeks	cc 128, minimally elevated protein	cMRI: restricted diffusion/FLAIR hyperintensity posterior splenium of corpus callosum	negative	oral prednisone 30 mg/d; later 1 mg/kg; methylpredn. i.v. 2mg/kg 5 days	major improvement
**Wilson et al [22]**	2018	35	male	Hodgkin lymphoma, relapsing	pembrolizumab	**LETM** (spastic tetraparesis, constipation, vomiting)	4 weeks	cc 24	LETM pons-lower thoracic spine	negative	methylprednisolone i.v.; plasma exchange	improvement
**Strik et al [23]**	2017	53	male	B-NHL	nivolumab	**encephalitis** (double vision, dysarthria, ataxia, mild cognitive dysfuntion)	not clear (patient reported with delay)	cc 15, protein 0.6-0.98 g/l	small FLAIR hyperintense, contrast-enhancing lesions periventr., midbrain, brainstem	negative	methylpredn. i.v. 1g/d 5 days; IVIG 1x; cyclophosphamide 2x 750 mg	symptoms stable but disabling; assisted suicide
**Garcia et al [24]**	2018	39	female	nodular melanoma	ipilimumab	**meningoencephalomyelitis** (headache, flu-like symptoms)	7 weeks	pleocytosis	brain MRI& **spinal** MRI: leptomeningeal enhancement; pituitary enlargement	negative	methylpredn. 1.0 mg/kg/d i.v.	rapid improvement; **relapse after 3 months** IVIG/steroid refractory: Infliximab 5mg/kg i.v. 3 doses: near complete recovery
**Naito et al [25]**	2018	57	male	SCLC	nivolumab + ipilimumab	**acute cerebellitis** (nystagmus, ataxia, dysarthria)	8 weeks	cc 35, protein 0.94g/l	marked cerebellar edema, diffuse FLAIR	n.d.	2x methylpredn. 1g/d 3 days, 6 cycles plasmapheres	limited improvement

									hyperintense cerebellar lesions		is; 2 cycles rituximab 375 mg/m2		
**Bot et al [26]**	2013	51	male	metastatic melanoma, cerebral metastasis	ipilimumab	**meningitis** (severe headache, fever)	3 weeks?	cc 20, protein 0.87 g/l	no new lesions	n.d.	dexamethasone p.o. 8 mg/d	complete recovery	
**Martinot et al [27]**	2018	54	male	Hodgkin lymphoma	nivolumab	**PML** (progressive hemiparesis left)	3 weeks after last course (14 months after start)	cc 1, **PCR: JCV 2230 c/ml**	multiple nonenhancing lesions	n.d.	none	no improvement	
**Matsuoka et al. [28]**	2018	60	male	pleomorphic lung carcinoma	nivolumab	**limbic encephalitis** (drosiness, muscular weakness, respiratory arrest)	5 weeks	cc 16, protein 1.62 g/l, OKB pos	T2 lesions temporal lobe, thalamus, aqueduct, spinal cord	anti-Hu AB positive (before Nivo)	high dose methylperdnisolone, 2x plasmapheresis	death	
**Chaucer et al [29]**	2018	44	male	metastatic renal cell carcinoma + HLRCC (autosomal dominant hereditary Leiomyomatosis and Renal Cell Carcinoma)	nivolumab	**encephalitis** (deteriorated mentation, hallucinations, aggressiveness)	2 weeks	n.d.	no metastases, no other lesions	n.d.	none	improvement	
**Patel et al. [30]**	2019	44	male	clear cell renal cell carcinoma	nivolumab; nivolumab + ipilimumab	non-infectious inflammatory process of the brain mimicking brain abcess	~ 9 weeks	n.d.	ring enhancing lesion in surgical cavity	n.d.	dexamethasone	complete recovery	nivolumab and ipilimumab restarted 5 weeks post-admission
**Quach et al [31]**	2019	n.a.	male	metastatic melanoma	pembrolizumab	**meningoencpehaltitis** (headache, altered mental	~7 weeks	inflammatory changes	n.a.	n.d.	high-dose steroids	complete recovery	

						status) + epididymo-Orchitis						
**Zafar et al [32]**	2019	59	female	laryngeal squamous cell carcinoma	nivolumab	**acute demyelinating encephalitis** (Nauseas, falls, weakness, altered mental status)	2 weeks	cc 74, elevated protein, OCB pos	multiple FLAIR hyperintense lesions parietal lobes + corpus callosum, Pons		methylprednisolone 1g/d 5 days, IVIG 20g/d 4 days	gradual improvement
**Shah et al [33]**	2018	66	female	lung adenocarcinoma	nivolumab	**encephalitis** (right hemiballismus, dysarthria)	16 weeks	cc normal, protein 0.56 mg/dl	T2-hyperintense basal ganglia abnormalities	novel, unclassified paraneoplastic AB (CSF)	methylprednisolone 1g/d 5 days; 5 PE; IVIG 2.5g/kg, rituximab 1000 mg 1x, tetrabenazine 20 mg (3x/d)	no improvement
		44	female	lung adenocarcinoma	nivolumab	**autoimmune encephalitis** (altered mental status, nausea, vomiting, seizure)	~9 weeks	cc 19, normal protein and glucose	T2 hyperintensities bilateral mesial temporal lobes + left occipital and right temporal lobe	GAD65-antibodies (CSF and serum)	methylprednisolone 1g/d 5 days; rituximab 1000 mg every 6 months	slight improvement
**Shibaki et al [34]**	2019	78	male	malignant pleura mesothelioma	nivolumab	**autoimmune encephalitis** (fever, somnolence, INO)	3 weeks	cc 15, protein 0.9 g/l	T2 hyperintensity mesencephalon and medial thalami (day 41)	anti-Ma2 AB positive	streoids	major improvement
**De la Hoz et al [35]**	2019	28	female	Hodgkin lymphoma	nivolumab	**autoimmune encephalitis** (headache, nausea, dizziness)	2 weeks	cc 136, protein 0.6 g/l	inconspicuous	n.d.	methylprednisolone 1mg/kg/d	complete recovery

**Kopecky et al [36]**	2018	63	male	metastatic renal cell carcinoma	nivolumab	**encephalopathy** (change in behavior, uncontrolled movements: choreatic)	12 weeks	mild inflammatory changes	symmetrical, increased singal in basal ganglia	anti-Ma2 AB positive (CSF)	methylprednisolone 2mg/kg/d; infliximab 5mg/kg	lost to follow-up	
**Leitinger et al [37]**	2018	67	female	squamous NSCLC	nivolumab	**necrotizing encephalopathy** (disorientation, speech arrest, apraxia, seizures)	4.5 weeks	cc 30, protein 0.56 g/l	first: unremarkable; follow-up: multiple, confluent FLAIR hyperintensities	negative	IVIG 30g/d, 5 days; methylprednisolone 1g/d 2 days	no improvement, death	neuropathological: beginning necrosis both thalami, left central region
**Läubli et al [38]**	2017	53	male	lung adenomcarcinoma	nivolumab	**cerebral vasculitis** (gait disturbance, speech difficulties)	not clear (soon after nivolumab initiation)	n.d.	new perietotemporal lesion	positive anti-SSA/Ro and anti-SSB/La AB	corticosteroids	improvement	histologically: necrotizing enecephalitis CD8+>CD4+ T cells
**Choe et al [39]**	2016	45	female	metastatic melanoma	ipilimumab	**meningoencephalitis** (confusion, dizziness, headache, myoclonic tremor)	6 weeks	cc 53, protein 1.51 g/l	no changes	negative	dexamethasone 8mg/d, methylperdnisolone 1mg(kg/d); IVIG 0.4g/kg/d 5 days	major improvement	
**Brown et al [40]**	2016	67	male	metastatic melanoma	pembrolizumab	**autoimmune limbic encephalitis** (short term memory loss, emotional lability, confusion)	~28 weeks	lymphocyctic pleocytosis	T2 hyperintensity medial temporal lobes + contrast enhancement	CASPR2 AB (CSF + serum) positive	methylprednisolone i.v.	improvement	
**Niki et al [41]**	2018	51	male	squamous NSCLC	pembrolizumab	**autoimmune encephalitis** (seizures, fever, headache, gait	24 weeks	cc 58, protein 4.46g/l	inconspicuous	negative	prednisolone 2mg/kg	improvement	

disturbances)

Table S2. Overview of published case-reports regarding peripheral nervous system manifestations as neurological adverse events of ICI-therapy. Besides patients' clinical data certain diagnostic findings as well as immunosuppressive.

author	year	patient age	patient sex	underlying diseases	ICI therapy	neurological symptoms	latency ICI-start/onset irAE	CSF findings	MRI	antibody findings	electrophysiology	immunosppr. therapy	outcome	additional information
**Wilson et al [22]**	2018	57	male	metastatic melanoma	nivolumab + ipilimumab	**myasthenia gravis**	4 weeks	unknown	unknown	negative		methylpredn. i.v.	rapid improvement	
**Johnson et al [42]**	2015	69	female	metastatic melanoma	ipilimumab	**myasthenia gravis**	around 10 weeks	n.d.	normal	Ach-R-antibodies positive (1,9 nmol/l)	decrement positive	methylpredn. i.v., plasmapheresis	improvement	
		73	female	metastatic melanoma	ipilimumab	**myasthenia gravis**	around 3 weeks	n.d.	n.d.	Ach-R-antibodies positive (13,6 nmol/l)	n.d.	high-dose corticosteroids	gradual improvement	
**Sciacca et al [43]**	2016	81	male	NSCLC	nivolumab	**myasthenia gravis**	4-5 weeks	n.d.	n.d.	Ach-R-antibodies 0,40 nmol/l	repetitive nerve stimulation negative; single fibre EMG of orbicularis oculi abnormal	prednisone	complete remission of MG	
**Shirai et al [44]**	2016	81	female	metastatic melanoma	nivolumab	**myasthenia gravis**	13 days	n.d.	n.d.	Ach-R-antibodies positive (12,4 mmol/l), others negative; CK	none	none	death	

										8729 U/l				
**Polat et al [45]**	2016	65	male	NSCLC	nivolumab	**myasthenia gravis**	6 weeks	n.d.	normal	negative	normal	pyridostigmine	continued improvement	
**Chang et al [46]**	2017	75	male	metastatic bladder SCC	nivolumab	**myasthenia gravis**	3 weeks	n.d.	normal	AchR-AB positive (2,28 nmol/l), CK 1587 U/l	significant decrement	IVIG	improvement	
**Loochtan et al [47]**	2017	70	male	SCLC	ipilimumab+nivolumab	**myasthenia gravis**	16 days	n.d.	n.d.	AchR-antibodies positive (1,64 mmol/l)	decrement	prednisolone p.o.; IVIG; plasmapheresis	death (bleeding, AV-blocking)	
**Derle et al [48]**	2018	71	male	metastatic melanoma	ipilimumab	**myasthenic crisis**	1 month	n.d.	n.d.	preexisting positive AchR-antibodies	n.d.	plasma exchange, IVIG, methylpredn.	death two years later due to respiratory failure	preexisting antibody positive MG, stable for 3 years under azathioprine 100 mg/d
**Montes et al [49]**	2018	74	male	metastatic melanoma	ipilimumab	**myasthenia gravis**	6 weeks	no abnormalities	n.d.	negative	decrement positive	high-dose corticosteroids	marked improvement	
**Lau et al [50]**	2016	75	male	metastatic melanoma	pembrolizumab	exacerbation of **myasthenia gravis**	4-5 weeks	n.d.	n.d.	n.d.	n.d.	methylpredn. i.v.; IVIG	marked improvement	preexisting antibody positive MG since 4 years, stable under Aza 200 mg/d
**Nguyen et al [51]**	2017	81	male	metastatic melanom	pembrolizumab	**myasthenia gravis**	11,5 weeks	n.d.	n.d.	negative	n.d.	prednisolone	complete	pembrolizumab was

				a									recovery	not stopped after ptosis
		86	female	metastatic melanoma	pembrolizumab	**myasthenia gravis**	6,5 weeks	no abnormalities	normal	negative	nerve conduction and repetitive stimulation normal/negative	methylpredn. i.v.	improvement	
**Alnahhas et al [52]**	2017	84	male	metastatic melanoma	pembrolizumab	**myasthenia gravis**	3 months	n.d.	n.d.	Ach-R-antibodies positive (0,05 nmol/l)	n.d.	prednisone 60 mg/d, pyridostigmine 60 mg 1-1-1; IVIG 0,4g/kg/d 5 days	death	
**Zhu et al [53]**	2016	59	female	metastatic melanoma	pembrolizumab	**myasthenia gravis**	around 11 weeks	n.d.	normal	negative	positive decrement	prednisone, IVIG, plasmapheresis	marked improvement	history of myasthenia gravis, rheumatoid arthritis, Ach-R-AB initially positive
**Gonzalez et al [54]**	2017	71	female	metastatic uterine carcinosarcoma	pembrolizumab	**myasthenia gravis**	after fourth dose (6-9 weeks)	n.d.	n.d.	CK 1200 U/l, antibodies negative	Single fiber EMG of the frontalis muscle abnormal	pyridostigmine + prednisone	significant improvement	
**Cooper et al [55]**	2017	68	female	NSCLC	nivolumab	**myasthenia gravis exacerbation**	12 weeks	n.d.	MRI brain + spinal cord: stable to minimally decreased size of known metastatic lesions	AChR 0.28 nmol/L	repetitive nerve stimulation: significant decrements	pyridostigmine, prednisone, plasmapheresis	death	preexisting anti-AChR antibody positive ocular MG
**Mehta et**	2017	73	mal	metastatic	nivolumab	**myasthenia**	18 days	no	n.d.	AchR-a		prednisolon	slight	

**al [56]**			e	renal cell ca		**gravis**		abnormalities		ntibodies positive (8,7 mmol/l); CK 8950 U/l		e + pyridostigmine, IVIG, plamapheresis	improvement	
**Becquart et al [57]**	2019	75	female	metastatic melanoma	nivolumab	**myasthenia gravis**	5 weeks	no abnormalities	brain MRI: normal	negative	normal	prostigmine	improvement	nivolumab continued for 1 more year
**Lara et al [58]**	2018	63	female	NSCLC	pembrolizumab	**myasthenia gravis**	~ 3 weeks	n.d.	brain MRI: unremarkable	highly elevated striatal muscle IgG antibody titer (1:1280).	n.d.	IVIG, high dose corticosteroid therapy, pyridostigmine	imrpovement	
**Werner et al [59]**	2019	62	male	metastatic melanoma	nivolumab + ipilimumab	**myasthenia gravis**	4 weeks	cc 13/μl	brain MRI: normal	negative	Repetitive stimulation: action potential decrement of 14%	pyridostigmine, prednisone	complete recovery	
**Nakatani et al [60]**	2018	73	female	squamous cell lung cancer	nivolumab	**Lambert-Eaton myasthenic syndrome**	20 weeks	n.d.	n.d.	anti-P/Q-type VGCC antibodies positive	low-amplitude compound muscle action potentials (CMAP), increasing >2 after brief exercise; positive repetitive nerve stimulation	pyridostigmine, ambenonium, ,4-DAP	gradual improvement	

**Kimura et al [61]**	2016	80	male	metastatic melanoma	nivolumab	**myasthenic crisis and polymoysitis**	2 weeks	n.d.	n.d.	AchR-antibodies positive (28 nmol/l) (before ICI: 10,2 nmol/l)	n.d.	methylpredn. i.v.; IVIG, plasma exchange	improvement	muscle biopsy: myositis
**March et al [62]**	2018	63	male	metastatic melanoma	pembrolizumab	**myasthenia gravis + myositis**	2 weeks	n.d.	normal (except for previously known metastases)	AchR-AB positive, CK 10286 U/l	n.d.	prednisone, pyridostigmine; IVIG; plasmapheresis	no improvement, death	
**Chen et al [63]**	2017	57	male	squamous cell carcinoma lung	nivolumab + ipilimumab	**coexisting myasthenia gravis, myositis, PNP**	4-6 weeks	no abnormalities	n.d.	AchR-antibodys slightly elevated	axonal polyneuropathy	prednisolone i.v. + pyridostigmine	improvement, death through infection	
**Liao et al [64]**	2014	70	female	metastatic uveal melanoma	ipilimumab	**myasthenia gravis + myositis**	6 weeks	n.d.	sMRI: degenerative disease	AchR-AB positive (2,09 nmol/l), CK 1200 U/l	diffuse myopathic findings; decrement positive	methylpredn. i.v., plasmapheresis; IVIG	improvement	
**Tan et al [65]**	2017	45	male	squamous cell carcinoma lung	nivolumab	**myasthenic crisis + myositis**	2 weeks	n.d.	brain MRI: normal	Ach-R-antibodies 2,0 nmol/l	no decrement	methylpredn. i.v.; IVIG	improvement	muscle biopsy: inflammatory myopathy
**Huh et al [66]**	2018	34	female	thymic cancer (Squamous cell carcinoma)	pembrolizumab	**myasthenic gravis + myositis**	unknown	n.d.	normal	Ach-R-antibodies 0,86 nmol/l; CK 2125 U/l	normal	IVIG , methylprednisolone, prednisolone; plasmapheresis	improvement	

**Konoeda et al [67]**	2017	74	female	advanced colon cancer	nivolumab	**myasthenic gravis + myositis**	3 weeks	n.a.	n.a.	n.a.	n.a.	methylpredn. i.v.; IVIG; plasma exchange	n.a.	
**Kang et al [68]**	2018	75	male	head and neck squamous cell carcinoma	nivolumab	**myositis and myasthenia gravis**	3 weeks	n.d.	n.d.	positive AChR-antibodies, anti-striated muscle antibodies 1:320; CK 2593 U/L	n.d.	methylpredn. i.v. , plasmapheresis, pyridostigmine	minimal improvement	
**So et al. [69]**	2019	55	female	metastatic melanoma	nivolumab	**severe myasthenia gravis + necrotizing myopathy and myocarditis**	2 weeks	n.d.	n.d.	positive AChR Ab (29 nmol/L), CK 13652 U/l	single-fiber electromyography: neuromuscular junction dysfunction (increased jitter)	steroid pulse, IVIG, plasma exchange	gradual improvement	AChR Ab (72 nmol/L) positive before nivolumab
**Moslehi et al [70]**	2017	65	female	metastatic melanoma	nivolumab + ipilimumab	**myocarditis + myositis**	12 days	n.d.	n.d.	antibodies n.d.; CK 17.720 U/l Trop I 51,3 ng/ml	n.d.	methylpredn. i.v.	death due to multisystem organ failure	postmortem cardiac and skeletal muscle biopsy: T-cell infiltration
		63	male	metastatic melanoma	nivolumab + ipilimumab	**myocarditis + myositis**	15 days	n.d.	n.d.	antibodies n.d.; CK 20.270 U/l,	n.d.	methylpredn.; Infliximab	death due to cardiac arrest	postmortem cardiac and skeletal muscle biopsy:

										Trop I 47 ng/ml)				T-cell infiltration
**Bourgeois-Vionnet et al [71]**	2018	79	male	metastatic lung adenocarcinoma	nivolumab	**myositis**	3 weeks	no abnormalities	sMRI: unremarkable	antibodies negative; CK 2450 U/L	Single fiber EMG and repetitive nerve stimulation negative	monthly IVIGs, oral corticosteroids	marked improvement	muscle biopsy: fascicular myonecrosis, phagocytosis, perivascular inflammatory infiltrates CD8+ T-cells
**Fox et al [72]**	2016	75	female	metastatic melanoma	nivolumab	**myositis**	2-3 weeks	n.d.	n.d.	antibodies n.d.; CK 1180 U/l	n.d.	prednisone	improvement	
**Saini et al [73]**	2017	35	male	Hodgkin lymphoma	nivolumab	**inflammatory myositis**	17 weeks	n.a.	n.a.	n.a.	n.a.	n.a.	n.a.	
**Badovinac et al [74]**	2018	64	female	squamous cell lung cancer	nivolumab	**inflammatory myositis**	28 weeks	n.d.	n.d.	CK 2657 U/l; antibodies negative	n.d.	methylpredn. i.v.	marked improvement	muscle biopsy: inflammatory origin of muscle fibers damage
**Pushkarevskaya et al [75]**	2017	60	female	metastatic melanoma	ipilimumab	**ocular myositis** + hypophysitis	about 17 weeks	3 normal lumbar punct.	brain MRI: preexisting brain metastases	TPO-AB positive, ANA 1:320; others negative (TRAK, SOX,	n.d.	methylpredn. i.v., mycophenolate; IVIG	marked improvement	

										VCCG)				
		60	female	metastatic melanoma	ipilimumab	**ocular myositis**	5 weeks	n.d.	orbital MRI: thickening of right superior oblique muscle; later: all other muscles	ANA 1:320, others negative	n.d.	methylpredn. i.v., mycophenolate	marked improvement	
**Lecouflet et al [76]**	2013	n.a.	female	metastatic melanoma	ipilimumab	**orbital myositis**	around 6-9 weeks	n.d.	orbital MRI: contrast enhancement left lateral rectus muscle	negative	n.d.	high-dose steroids oral	complete recovery	
**Sheik et al [77]**	2015	55	female	metastatic melanoma	ipilimumab	**dermatomyositis**	2 weeks; relapse after 14 months	n.d.	n.d.	ANA 1:640, others negative (Jo-1..); CK 1854 U/l	n.d.	prednisone; methylpredn. i.v.	marked improvement of muscle strength	muscle biopsy: atrophy but no inflammatory myositis
**Yoshioka et al [78]**	2015	84	male	metastatic melanoma	nivolumab	**myositis**	7 weeks	n.d.	n.d.	antibodies n.d.; CK 2812 U/l	n.d.	prednisolone	complete recovery	
**Vallet et al [79]**	2016	86	female	metastatic melanoma	pembrolizumab	**necrotic myositis**	3,5 weeks	n.d.	n.d.	negative	EMG: myopathic changes	methylpredn. i.v., plasma exchange	complete recovery	muscle biopsy: multifocal necrosis, endomysial CD8+ infiltrates
**Haddox et al [80]**	2017	78	male	metastatic melanom	pembrolizumab	bulbar myopathy,	5 weeks	n.d.	sMRI: symetric	striational	EMG: MUAP	prednisone, plasma	death	muscle biopsy:

				a		**necrotizing myositis** of diaphragm			enhancement of paraspinal muscles	antibodies 1:61440; others negative; CK 1284 U/l	amplitudes reduced, fibrillation potentials in prox. muscle groups and orbicularis oculi	exchange		necrotic fibres in most fascicles; autopsy: difuse necrotic myositis of diaphragm, lymphohistiocytic myocarditis
**Bilen et al [81]**	2016	73	male	metastatic transitional cell carcinoma	nivolumab + ipilimumab	**rhabdomyolysis + polymyositis**	3,5 weeks	n.d.	n.d.	anti-SM-AB 1:61440; CK 13710 U/l	n.d.	methylpredn.; infliximab; IVIG; plasmapheresis	slight improvement	
**Gandiga et al [82]**	2018	44	female	metastatic melanoma	pembrolizumab	**inflammatory myopathy**	2 months	n.d.	n.d.	negative; CK 2395 U/l	EMG: duration and amplitude reduced, increased PSA	methylpredn. i.v. + IVIG	death due to progressive melanoma	preexisting seropositive RA; muscle biopsy: inflammatory infiltrates, necrotic muscle fibres
**Diamantopoulos et al [83]**	2017	82	male	melanoma	pembrolizumab	**inflammatory myopathy + axonal neuropathy**	15 days	normal	n.d.	negative; CK 4670 U/l	EMG: short-duration, small-amplitude motor unit potentials, PSA	prednisolone i.v. + IVIG, plasmapheresis	death (respiratory failure)	muscle biopsy: scrace straited muscl tissue, insufficient quality
**Min et al [84]**	2014	46	male	metastatic melanoma	ipilimumab	**rhabdomyolysis+** hypothyroidism	14-15 weeks	n.d.	n.d.	n.d.; CK 30.980 U/l	n.d.	none; (L-thyroxine)	complete recovery	

**Carrera et al [85]**	2017	68	male	NSCLC	tremelimumab + durvalumab	**myopathy involving extraocular muscles**	1 month	n.d.	brain MRI: normal	antibodies negative; CK 3083 U/l	EMG: complex polyphasic motor unit potentials -->inflammat. myopathy	prednisone	improvement	muscle biopsy: infiltrat. Of endomysium and perimysium by CD4-, CD8+ cells
**Kadota et al. [86]**	2019	85	female	metastatic melanoma	nivolumab	**dermatomyositis**	4 weeks	n.d.	n.d.	negative	n.d.	prednisolone	improvement	
**Ogawa et al. [87]**	2017	88	male	metastatic melanoma	nivolumab	**polymyositis**	6 weeks	n.d.	n.d.	negative	EMG: myogenic pattern	oral prednisolone, azathioprine	death	
**Sekiguchi et al. [88]**	2019	78	male	bladder cancer	pembrolizumab	**myositis + diaphragm involvement**	2 weeks	n.d.	n.d.	AChR-AB positive 9.5 nmol/l; anti-striational muscle antibodies positive; CK 2015 U/l	no decrement in repetitive stimulation	methylpredn. i.v., IVIG	partial improvement (limb muscle weakness recovered but respiratory function did not)	
		52	male	metastatic melanoma	nivolumab + ipilimumab	**GBS**	3 -4 weeks	protein 2.3 g/l	inconspicuous	negative		IVIG	very good recovery	
**Garcia et al [24]**	2018	55	male	melanoma IIIB	ipilimumab	**acute infl. demyelinating PNP**	6 weeks	lymphocytic pleocytosis, protein 1.75 g/l	abnormal enhancement bilat. 5th, 7th, 8th cranial nerves, cauda equina, conus	negative		methylpredn. i.v.	rapid motor improvement; minimal paresthesia continued	
**Bot et al**	2013	63	mal	metastatic	ipilimumab	**axonal GBS**	about 12	cc normal,	normal			IVIG	died	

**[26]**			e	melanoma			weeks (3weeks after 4th course)	protein 0.89 g/l					from respiratory insufficiency
**Ong et al [89]**	2018	66	male	lung adenocarcinoma	pembrolizumab	**GBS**	1 month		spinal MRI: only degenerative changes	negative	tibial DML prolonged, partial conduction block peroneus bilateral, sparing of sural sensory response	methylpredn. i.v.; IVIG	almost full recovery
**De Maleissye et al [90]**	2016	45	female	melanoma	pembrolizumab	**GBS**	8 weeks	slight pleocytosis, protein 0.56 g/l	normal	negative	multifocal demylination with conduction blocks	prednisolone, IVIG	improvement
**Schneiderbauer et al [91]**	2017	51	male	metastatic melanoma	nivolumab	**GBS**	5 months	cc normal, protein 0.73 g/l	brain MRI and sMRI: normal	negative	acute demyelinating sensorimotor PNP	methylpredn. i.v. + IVIG	nearly complete recovery
**Fukumoto et al [92]**	2018	66	male	metastatic NSCLC	nivolumab	**acute demyelinating polyneuropathy**	3 weeks	cc 4/µl, protein 3.4 g/l	n.d.	GM2-+Ga1Nac-GD1a-IgM-antibodies	DML prolonged, NCV reduced	prednisolone; IVIG	improvement
**Supakornnumporn et al [93]**	2017	77	male	metastatic melanoma	ipilimumab + nivolumab	**GBS**	7 weeks	cc normal, protein 0.86 g/l	n.d.	n.d.	reduced median and ulnar cMAP amplitudes, median and ulnar conduction blocks in forearm	prednisone, IVIG	improvement
**Yost et**	2017	64	mal	melanom	ipilimumab +	**GBS**	17	cc 12/µl,	subtle	negativ	mildly slow	IVIG,	marked

**al [94]**			e	a	pembrolizumab		months ??	protein 1.95 g/l	enhancement of facial nerves	e	lower limb motor conduction velocities, pathological blink reflexes	prednisone p.o.	improvement
**Jacob et al [95]**	2016	68	female	metastatic squamous cell carcinoma lung	nivolumab	**GBS**	3 months	cc normal, protein 0.85 g/l	sMRI: normal	n.d.	n.d.	IVIG + plasmapheresis	death (respiratory failure)
**Wilgenhof et al [96]**	2011	57	female	metastatic melanoma	ipilimumab	**GBS**	7 weeks	cc normal, protein 1.67 g/l	brain MRI + sMRI: nomal	n.d.	motor and sensory demyelinating PNP	methylpredn. i.v.	nearly complete recovery
**Kelly Wu et al [97]**	2017	37	female	metastatic melanoma	ipilimumab	**GBS**	unknown	unknown	n.d.	negative	f-waves prolonged, amplitudes smaller	Droxidopa, IVIG	slight improvement
**Gu et al [98]**	2017	49	female	metastatic melanoma	ipilimumab + nivolumab	**acute neuropathy**	5 days	cc: 15/µl, protein 1.15 g/l	spinal MRI: normal	negative	acute generalized motor predominant neuropathy	IVIG, methylpredn. i.v.; mycophenolate + plasma exchange	mild improvement; relapse after 1 month
		81	male	metastatic melanoma (brain metastases)	pembrolizumab	**acute inflammatory demyelinating PNP**	4 weeks (after 2nd cycle)	albuminocytol. dissociation with elevated CSF protein	brain MRI: brain metastases	negative	consistent with motor and sensory neuropathy	methylpredn., IVIG, plasmapheresis	death (hemorrhage within brain metastasis)
**Nukui et al. [99]**	2018	45	male	nasal cancer	nivolumab	**acute demyelinating polyradiculoneuropathy**	~ 10 weeks	cc 7/µl, protein 3.5 g/l	brain MRI: not remarkable; spinal MRI: gadolinium enhancement of nerve roots and cauda	negative	prolonged distal latency (DL), reduced nerve conduction velocity (NCV)	IVIG, steroid pulse therapy	improvement

									equina					
		85	female	metastatic melanoma	ipilimumab + pembrolizumab	**CIDP**	20 weeks	cc normal, protein 0.74 g/l	normal	negative	multifocal demylination with conduction blocks	oral and i.v. glucocorticoids, plasma exchange	no improvement	
		44	male	metastatic melanoma	ipilimumab	**CIDP**	1 week	cc normal, protein 0.44 g/l	normal (except for previously known metastases)	negative	all potentials of blink response markedly delayed (demyelinating PNP)	plasmapheresis	significant improvement	
**Thaipisuttikul et al [100]**	2015	57	male	metastatic melanoma	ipilimumab	**peripheral neuropathy**	36 days	**1.** cc 78/µl, protein 0.68 g/l **2.** cc 79/µl, protein 0.95 g/l **3.** cc 8/µl, protein 0.32 g/l	brain MRI + sMRI: nomal	negative	symetric sensorimotor PNP	methylpredn.; infliximab; IVIG; tacrolimus + methylpredn.	slight improvement	
**Tanaka et al [101]**	2016	85	female	metastatic melanoma	nivolumab	**CIDP**	2 weeks	cc 11/µl, protein 3.58 g/l	brain MRI: normal; sMRI: gadolinium enhancement C7 and Th1 dorsal roots	negative	F-wave latency prolonged, conduction blocks	prednisolone; IVIG	improvement	suralis biopsy: loss of small myelinated fibres, mild lymphocytic infiltration
**Sepulveda et al [102]**	2017	44	male	metastatic melanoma	pembrolizumab	**motor polyradiculopathy**	47 weeks	cc normal, protein 0.67 g/l	brain MRI: normal; sMRI: diffuse enhancement of dorsal roots	negative	ENG: normal, EMG: reduction of number of active motor unitpotentials	IVIG + oral prednisone; plasma exchange	gradual improvement	
**Aya et al**	2017	53	fem	metastatic	ipilimumab;	**vasculitic**	2 weeks	n.d.	n.d.	n.d.	moderate	methylpredn	slow	preexisting

**[103]**			ale	melanoma	pembrolizumab	**neuropathy**					sensory PNP	. i.v., oral prednisolone	improvement	seropositive RA; muscle biopsy: perivascular infiltration (small endoneural vessels) of mononuclear cells
**Manam et al [104]**	2018	73	male	adenocarcinoma of the lung	pembrolizumab	**inflammatory demyelinating PNP**	3 weeks	albuminocytol. dissociation with elevated CSF protein	n.d.	negative	n.d.	methylpredn., IVIG, plamaspheresis	gradual improvement	
**Simsek et al. [105]**	2018	52	male	metastatic renal cell carcinoma	nivolumab	**peripheral neuropathy**	10 weeks	n.d.	brain MRI and sMRI: unremarkable	CK 2440 U/l	n.d.	methylpredn. i.v.	improvement	
		62	male	metastatic melanoma	ipilimumab	**peripheral neuropathy**	13 weeks	n.d.	n.d.	n.d.	n.d.	methylpredn .i.v. + mycophenolate	full improvement	
**Sakai et al [106]**	2017	81	male	metastatic melanoma	nivolumab	**mononeuropathy multiplex + rhabdomyolysis**	8 days	cc 1/µl, protein 0.27 g/l	n.d.	negative (CK 27703 U/l)	axonal mononeuropathy multiplex	prednisolone i.v.	improvement	
**Zecchini et al. [107]**	2018	45	male	metastatic melanoma	ipilimumab + nivolumab, followed by nivolumab mono	**Bell's palsy**	2 weeks	cc 240/µl, protein 0.26 g/l	brain MRI: unremarkable	n.d.	n.d.	oral valacyclovir + oral prednisone	complete resolution	
**Ziemann et al [108]**	2019	51	female	metastatic melanoma	ipilimumab + nivolumab	**Bell's palsy + polyneuropathy**	1 week	n.d.	brain MRI: normal	n.d.	n.d.	prednisone taper and acyclovir	improvement	
		68	female	metastatic melanoma	ipilimumab + nivolumab	**Bell's palsy**	about 3 weeks	n.d.	brain MRI: normal	n.d.	n.d.	prednisone taper + valacyclovir.	improvement	

**Jinnur et al [109]**	2015	66	male	metastatic melanoma	ipilimumab	**bilateral phrenic nerve neuropathy**	about 12 weeks	n.d.	sMRI normal	negative	bilateral severe phrenic nerve neuropathies	prednisone	no improvement (needed BiPAP during sleep)	
**Ghosn et al [110]**	2018	69	female	metastatic melanoma	pembrolizumab (+ T-VEC)	**sjögren's syndrome**	20 weeks	cc 92 /µl, protein 1.32 g/l,	brain MRI: enhancement of the right trigeminal Gasser's ganglia and its mandibular branch	anti-SSA elevated, other antibodies negative	absence of SNAPs in upper and lower limbs; reduced MAPs for the median and ulnar nerves	methylpredn. i.v.; IVIGs; 2nd line cyclophosphamide (replaced ny rituximab)+ oral prednisone	improvement (but residuals)	biopsy of accessory salivary glands (ASGB): abnormal interstitial sclerosis with a focus of > 50 lymphocytes/4 mm2 (Chisholm and Mason's score of 3 out of 4)

References

1. Bossart, S.; Thurneysen, S.; Rushing, E.; Frontzek, K.; Leske, H.; Mihic-Probst, D.; Nagel, H.W.; Mangana, J.; Goldinger, S.M.; Dummer, R. Case Report: Encephalitis, with Brainstem Involvement, Following Checkpoint Inhibitor Therapy in Metastatic Melanoma. *Oncologist* **2017**, *22*, 749–753.
2. Burke, M.; Hardesty, M.; Downs, W. A case of severe encephalitis while on PD-1 immunotherapy for recurrent clear cell ovarian cancer. *Gynecol. Oncol. Rep.* **2018**, *24*, 51–53.
3. Maurice, C.; Schneider, R.; Kiehl, T.-R.; Bavi, P.; Roehrl, M.H.A.; Mason, W.P.; Hogg, D. Subacute CNS Demyelination after Treatment with Nivolumab for Melanoma. *Cancer Immunol. Res.* **2015**, *3*, 1299–1302.
4. Richard, K.; Weslow, J.; Porcella, S.L.; Nanjappa, S. A Case Report of Steroid Responsive Nivolumab-Induced Encephalitis. *Cancer Control.* **2017**, *24*, 1073274817729069.
5. Schneider, S.; Potthast, S.; Komminoth, P.; Schwegler, G.; Böhm, S. PD-1 Checkpoint Inhibitor Associated Autoimmune Encephalitis. *Case Rep. Oncol.* **2017**, *10*, 473–478.
6. Williams, T.J.; Benavides, D.R.; Patrice, K.-A.; Dalmau, J.O.; De Ávila, A.L.R.; Le, D.T.; Lipson, E.J.; Probasco, J.C.; Mowry, E.M. Association of Autoimmune Encephalitis With Combined Immune Checkpoint Inhibitor Treatment for Metastatic Cancer. *JAMA Neurol.* **2016**, *73*, 928.
7. Kawamura, R.; Nagata, E.; Mukai, M.; Ohnuki, Y.; Matsuzaki, T.; Ohiwa, K.; Nakagawa, T.; Kohno, M.; Masuda, R.; Iwazaki, M.; et al. Acute Cerebellar Ataxia Induced by Nivolumab. *Intern. Med.* **2017**, *56*, 3357–3359.
8. Narumi, Y.; Yoshida, R.; Minami, Y.; Yamamoto, Y.; Takeguchi, S.; Kano, K.; Takahashi, K.; Saito, T.; Sawada, J.; Terui, H.; et al. Neuromyelitis optica spectrum disorder secondary to treatment with anti-PD-1 antibody nivolumab: The first report. *BMC Cancer* **2018**, *18*, 95.
9. O'Kane, G.M.; Lyons, T.G.; Colleran, G.C.; Alken, S.; Kavanagh, E.C.; Ahmad, M.W.; Fitzpatrick, D.; Murray, B.; Kelly, C.M. Late-Onset Paraplegia after Complete Response to Two Cycles of Ipilimumab for Metastatic Melanoma. *Oncol. Res. Treat.* **2014**, *37*, 757–760.
10. Tan, I.; Malinzak, M.; Salama, A.K.S. Delayed onset of neurosarcoidosis after concurrent ipilimumab/nivolumab therapy. *J. Immunother. Cancer* **2018**, *6*, 77.
11. Salam, S.; Lavin, T.; Turan, A. Limbic encephalitis following immunotherapy against metastatic malignant melanoma. *BMJ Case Rep.* **2016**, *2016*, doi:10.1136/bcr-2016-215012.
12. Stein, M.K.; Summers, B.B.; Wong, C.A.; Box, H.L.; Cleveland, K.O. Meningoencephalitis Following Ipilimumab Administration in Metastatic Melanoma. *Am. J. Med. Sci.* **2015**, *350*, 512–513.
13. Khoja, L.; Maurice, C.; Chappell, M.; Macmillan, L.; Al-Habeeb, A.S.; Al-Faraidy, N.; Butler, M.O.; Rogalla, P.; Mason, W.; Joshua, A.M.; et al. Eosinophilic Fasciitis and Acute Encephalopathy Toxicity from Pembrolizumab Treatment of a Patient with Metastatic Melanoma. *Cancer Immunol. Res.* **2016**, *4*, 175–178.
14. Gettings, E.J.; Hackett, C.T.; Scott, T.F. Severe relapse in a multiple sclerosis patient associated with ipilimumab treatment of melanoma. *Mult. Scler.* **2015**, *21*, 670.
15. LaPorte, J.; Solh, M.; Ouanounou, S. Posterior reversible encephalopathy syndrome following pembrolizumab therapy for relapsed Hodgkin's lymphoma. *J. Oncol. Pharm. Pract.* **2017**, *23*, 71–74.
16. Maur, M.; Tomasello, C.; Frassoldati, A.; Dieci, M.V.; Barbieri, E.; Conte, P. Posterior Reversible Encephalopathy Syndrome During Ipilimumab Therapy for Malignant Melanoma. *J. Clin. Oncol.* **2012**, *30*, e76–e78.
17. Mandel, J.J.; Olar, A.; Aldape, K.D.; Tremont-Lukats, I.W. Lambrolizumab induced central nervous system (CNS) toxicity. *J. Neurol. Sci.* **2014**, *344*, 229–231.
18. Bompaire, F.; Mateus, C.; Taillia, H.; De Greslan, T.; Lahutte, M.; Sallansonnet-Froment, M.; Ouologuem, M.; Renard, J.L.; Gorochov, G.; Robert, C.; et al., Severe meningo-radiculo-neuritis associated with ipilimumab. *Invest. New Drugs* **2012**, *30*, 2407–2410.
19. Altman, A.L.; Golub, J.S.; Pensak, M.L.; Samy, R.N. Bilateral Facial Palsy following Ipilimumab Infusion for Melanoma. *Otolaryngol. Head Neck Surg.* **2015**, *153*, 894–895.
20. Carl, D.; Grüllich, C.; Hering, S.; Schabet, M. Steroid responsive encephalopathy associated with autoimmune thyroiditis following ipilimumab therapy: A case report. *BMC Res. Notes* **2015**, *8*, 316.
21. Conry, R.M.; Sullivan, J.C.; Nabors, and L.B.3rd. Ipilimumab-induced encephalopathy with a reversible splenial lesion. *Cancer Immunol. Res.* **2015**, *3*, 598–601.

22. Wilson, R.; Menassa, D.A.; Davies, A.J.; Michael, S.; Hester, J.; Kuker, W.; Collins, G.P.; Cossins, J.; Beeson, D.; Steven, N.; et al. Seronegative antibody-mediated neurology after immune checkpoint inhibitors. *Ann. Clin. Transl. Neurol.* **2018**, *5*, 640–645.
23. Strik, H.; Keber, U.; Hammoud, W.A.; Riera-Knorrenschild, J.; Carl, B.; Dodel, R.; Pagenstecher, A.; Neubauer, A. Immune checkpoint inhibitor–associated CNS autoimmune disorder (ICICAD) following nivolumab treatment: A new entity of drug-induced autoimmune encephalitis?. *Eur. J. Cancer* **2017**, *87*, 205–208.
24. Garcia, C.A.; El-Ali, A.; Rath, T.J.; Contis, L.C.; Gorantla, V.; Drappatz, J.; Davar, D. Neurologic immune-related adverse events associated with adjuvant ipilimumab: Report of two cases. *J. Immunother. Cancer* **2018**, *6*, 83.
25. Naito, T.; Osaki, M.; Ubano, M.; Kanzaki, M.; Uesaka, Y. Acute cerebellitis after administration of nivolumab and ipilimumab for small cell lung cancer. *Neurol. Sci.* **2018**, *39*, 1791–1793.
26. Bot, I.; Blank, C.U.; Boogerd, W.; Brandsma, D. Neurological immune-related adverse events of ipilimumab. *Pract. Neurol.* **2013**, *13*, 278–280.
27. Martinot, M.; Ahle, G.; Petrosyan, I.; Martinez, C.; Gorun, D.M.; Mohseni-Zadeh, M.; Fafi-Kremer, S.; Tebacher-Alt, M. Progressive Multifocal Leukoencephalopathy after Treatment with Nivolumab. *Emerg. Infect. Dis.* **2018**, *24*, 1594–1596.
28. Matsuoka, H.; Kimura, H.; Koba, H.; Tambo, Y.; Ohkura, N.; Hara, J.; Sone, T.; Kasahara, K. Nivolumab-induced Limbic Encephalitis with Anti-Hu Antibody in a Patient With Advanced Pleomorphic Carcinoma of the Lung. *Clin. Lung Cancer* **2018**, *19*, e597–e599.
29. Chaucer, B.; Stone, A.; Demanes, A.; Seibert, S.M. Nivolumab-Induced Encephalitis in Hereditary Leiomyomatosis and Renal Cell Cancer Syndrome. *Case Rep. Oncol. Med.* **2018**, *2018*, 4273231, doi:10.1155/2018/4273231.
30. Patel, A.R.; Connors, S.; Wardak, Z.; Brugarolas, J.; Patel, T.R. Inflammatory Reaction Secondary to Immune Checkpoint Inhibitor Therapy Mimicking a Post-Operative Brain Abscess. *World Neurosurg.* **2019**, *129*, 354–358.
31. Quach, H.T.; Robbins, C.J.; Balko, J.M.; Chiu, C.Y.; Miller, S.; Wilson, M.R.; Nelson, G.E.; Johnson, D.B. Severe Epididymo-Orchitis and Encephalitis Complicating Anti-PD-1 Therapy. *Oncologist* **2019**, *24*, 872–876.
32. Zafar, Z.; Vogler, C.; Hudali, T.; Bhattarai, M. Nivolumab-Associated Acute Demyelinating Encephalitis: A Case Report and Literature Review. *Clin. Med. Res.* **2019**, *17*, 29–33.
33. Shah, S.; Dunn-Pirio, A.; Luedke, M.; Morgenlander, J.; Skeen, M.; Eckstein, C. Nivolumab-Induced Autoimmune Encephalitis in Two Patients with Lung Adenocarcinoma. *Case Rep. Neurol. Med.* **2018**, *2018*, 2548528, doi:10.1155/2018/2548528.
34. Shibaki, R.; Murakami, S.; Oki, K.; Ohe, Y. Nivolumab-induced autoimmune encephalitis in an anti-neuronal autoantibody-positive patient. *Jpn. J. Clin. Oncol.* **2019**, *49*, 793–794.
35. De la Hoz, A.; Foolad, F.; Gallegos, C.; Kornblau, S.; Kontoyiannis, D.P. Nivolumab-induced encephalitis post allogeneic stem cell transplant in a patient with Hodgkin's disease. *Bone Marrow Transplant.* **2019**, *54*, 749–751.
36. Kopecký, J.; Kubeček, O.; Geryk, T.; Slováčková, B.; Hoffmann, P.; Žiaran, M.; Priester, P. Nivolumab induced encephalopathy in a man with metastatic renal cell cancer: A case report. *J. Med. Case Rep.* **2018**, *12*, 262.
37. Leitinger, M., Varosanec, M.V.; Pikija, S.; Wass, R.E.; Bandke, D.; Weis, S.; Studnicka, M.; Grinzinger, S.; McCoy, M.R.; Hauer, L.; et al., Fatal Necrotizing Encephalopathy after Treatment with Nivolumab for Squamous Non-Small Cell Lung Cancer: Case Report and Review of the Literature. *Front. Immunol.* **2018**, *9*, 108.
38. Läubli, H.; Hench, J.; Stanczak, M.; Heijnen, I.; Papachristofilou, A.; Frank, S.; Zippelius, A.; Stenner-Liewen, F. Cerebral vasculitis mimicking intracranial metastatic progression of lung cancer during PD-1 blockade. *J. Immunother. Cancer* **2017**, *5*, 46.
39. Choe, J.H.; Andonian, B.J.; Kim, G.J.; Salama, A.K. Autoimmune meningoencephalitis in a melanoma patient treated with ipilimumab. *Immunotherapy* **2016**, *8*, 1163–1167.
40. Brown, M.P.; Hissaria, P.; Hsieh, A.H.; Kneebone, C.; Vallat, W. Autoimmune limbic encephalitis with anti-contactin-associated protein-like 2 antibody secondary to pembrolizumab therapy. *J. Neuroimmunol.* **2017**, *305*, 16–18.

41. Niki, M., Nakaya, A.; Kurata, T.; Nakahama, K.; Yoshioka, H.; Kaneda, T.; Kibata, K.; Ogata, M.; Nomura, S. Pembrolizumab-induced autoimmune encephalitis in a patient with advanced non-small cell lung cancer: A case report. *Mol. Clin. Oncol.* **2019**, *10*, 267–269.
42. Johnson, D.B., Saranga-Perry, V.; Lavin, P.J.; Burnette, W.B.; Clark, S.W.; Uskavitch, D.R.; Wallace, D.E.; Dickson, M.A.; Kudchadkar, R.R.; Sosman, J.A. Myasthenia Gravis Induced by Ipilimumab in Patients With Metastatic Melanoma. *J. Clin Oncol* **2015**, *33*, e122–e124.
43. Sciacca, G.; Nicoletti, A.; Rampello, L.; Noto, L.; Parra, H.J.S.; Zappia, M. Benign form of myasthenia gravis after nivolumab treatment. *Muscle Nerve* **2016**, *54*, 507–509.
44. Shirai, T., Sano, T.; Kamijo, F.; Saito, N.; Miyake, T.; Kodaira, M.; Katoh, N.; Nishie, K.; Okuyama, R.; Uhara, H. Acetylcholine receptor binding antibody-associated myasthenia gravis and rhabdomyolysis induced by nivolumab in a patient with melanoma. *Jpn. J. Clin. Oncol.* **2016**, *46*, 86–88.
45. Polat, P.; Donofrio, P.D. Myasthenia gravis induced by nivolumab therapy in a patient with non-small-cell lung cancer. *Muscle Nerve* **2016**, *54*, 507.
46. Chang, E.; Sabichi, A.L.; Sada, Y.H. Myasthenia Gravis After Nivolumab Therapy for Squamous Cell Carcinoma of the Bladder. *J. Immunother.* **2017**, *40*, 114–116.
47. Earl, D.E.; Loochtan, A.I.; Bedlack, R.S. Refractory myasthenia gravis exacerbation triggered By pembrolizumab. *Muscle Nerve* **2018**, *57*, E120–E121.
48. Derle, E.; Benli, S. Ipilimumab treatment associated with myasthenic crises and unfavorable disease course. *Neurol. Sci.* **2018**, *39*, 1773–1774.
49. Montes, V.; Sousa, S.; Pita, F.; Guerreiro, R.; Carmona, C. Myasthenia Gravis Induced by Ipilimumab in a Patient With Metastatic Melanoma. *Front. Neurol.* **2018**, *9*, 150.
50. Lau, K.H.V.; Kumar, A.; Yang, I.H.; Nowak, R.J. Exacerbation of myasthenia gravis in a patient with melanoma treated with pembrolizumab. *Muscle Nerve* **2016**, *54*, 157–161.
51. Nguyen, B.H.; Kuo, J.; Budiman, A.; Christie, H.; Ali, S. Two cases of clinical myasthenia gravis associated with pembrolizumab use in responding melanoma patients. *Melanoma Res.* **2017**, *27*, 152–154.
52. Alnahhas, I.; Wong, J. A case of new-onset antibody-positive myasthenia gravis in a patient treated with pembrolizumab for melanoma. *Muscle Nerve* **2017**, *55*, E25–E26.
53. Zhu, J.; Li, Y. Myasthenia gravis exacerbation associated with pembrolizumab. *Muscle Nerve* **2016**, *54*, 506–507.
54. Gonzalez, N.L.; Puwanant, A.; Lu, A.; Marks, S.M.; Živković, S.A. Myasthenia triggered by immune checkpoint inhibitors: New case and literature review. *Neuromuscul. Disord.* **2017**, *27*, 266–268.
55. Cooper, D.S.; Meriggioli, M.N.; Bonomi, P.D.; Malik, R. Severe Exacerbation of Myasthenia Gravis Associated with Checkpoint Inhibitor Immunotherapy. *J. Neuromuscul. Dis.* **2017**, *4*, 169–173.
56. Mehta, J.J.; Maloney, E.; Srinivasan, S.; Seitz, P.; Cannon, M. Myasthenia Gravis Induced by Nivolumab: A Case Report. *Cureus* **2017**, *9*, e1702.
57. Becquart, O.; Lacotte, J.; Malissart, P.; Nadal, J.; Lesage, C.; Guillot, B.; Du Thanh, A.; Julie, L.; Pauline, M.; Jeremy, N.; et al. Myasthenia Gravis Induced by Immune Checkpoint Inhibitors. *J. Immunother.* **2019**, *42*, 309–312.
58. Lara, M.S.; Afify, A.; Ellis, M.P.; Phan, C.T.; Richman, D.P.; Riess, J.W. Immune Checkpoint Inhibitor-Induced Myasthenia Gravis in a Patient with Advanced NSCLC and Remote History of Thymoma. *Clin. Lung Cancer* **2019**, *20*, e489–e491.
59. Werner, J.-M.; Schweinsberg, V.; Schroeter, M.; Von Reutern, B.; Malter, M.P.; Schlaak, M.; Fink, G.R.; Mauch, C.; Galldiks, N. Successful Treatment of Myasthenia Gravis Following PD-1/CTLA-4 Combination Checkpoint Blockade in a Patient With Metastatic Melanoma. *Front. Oncol.* **2019**, *9*, 84.
60. Nakatani, Y.; Tanaka, N.; Enami, T.; Minami, S.; Okazaki, T.; Komuta, K. Lambert-Eaton Myasthenic Syndrome Caused by Nivolumab in a Patient with Squamous Cell Lung Cancer. *Case Rep. Neurol.* **2018**, *10*, 346–352.
61. Kimura, T.; Fukushima, S.; Miyashita, A.; Aoi, J.; Jinnin, M.; Kosaka, T.; Ando, Y.; Matsukawa, M.; Inoue, H.; Kiyotani, K.; et al. Myasthenic crisis and polymyositis induced by one dose of nivolumab. *Cancer Sci.* **2016**, *107*, 1055–1058.
62. March, K.L.; Samarin, M.J.; Sodhi, A.; Owens, R.E. Pembrolizumab-induced myasthenia gravis: A fatal case report. *J. Oncol. Pharm. Pract.* **2018**, *24*, 146–149.

63. Chen, J.H., Lee, K.Y.; Hu, C.J.; Chung, C.C. Coexisting myasthenia gravis, myositis, and polyneuropathy induced by ipilimumab and nivolumab in a patient with non-small-cell lung cancer: A case report and literature review. *Medicine (Baltimore)* **2017**, *96*, e9262.
64. Liao, B.; Shroff, S.; Kamiya-Matsuoka, C.; Tummala, S. Atypical neurological complications of ipilimumab therapy in patients with metastatic melanoma. *Neuro. Oncol.* **2014**, *16*, 589–593.
65. Tan, R.Y.C.; Toh, C.K.; Takano, A. Continued Response to One Dose of Nivolumab Complicated by Myasthenic Crisis and Myositis. *J. Thorac. Oncol.* **2017**, *12*, e90–e91.
66. Huh, S.Y.; Shin, S.H.; Kim, M.K.; Lee, S.Y.; Son, K.H.; Shin, H.Y. Emergence of Myasthenia Gravis with Myositis in a Patient Treated with Pembrolizumab for Thymic Cancer. *J. Clin. Neurol.* **2018**, *14*, 115–117.
67. Konoeda, F.; Suzuki, S.; Nishimoto, Y.; Hoshino, H.; Takagi, M. A case of myasthenia gravis and myositis induced by nivolumab. *Rinsho Shinkeigaku* **2017**, *57*, 373–377.
68. Kang, K.H.; Grubb, W.; Sawlani, K.; Gibson, M.K.; Hoimes, C.J.; Rogers, L.R.; Lavertu, P.; Yao, M. Immune checkpoint-mediated myositis and myasthenia gravis: A case report and review of evaluation and management. *Am. J. Otolaryngol.* **2018**, *39*, 642–645.
69. So, H.; Ikeguchi, R.; Kobayashi, M.; Suzuki, M.; Shimizu, Y.; Kitagawa, K. PD-1 inhibitor-associated severe myasthenia gravis with necrotizing myopathy and myocarditis. *J. Neurol. Sci.* **2019**, *399*, 97–100.
70. Moslehi, J.J.; Johnson, D.B.; Sosman, J. A.; Caio, G. Myocarditis with Immune Checkpoint Blockade. *New Engl. J. Med.* **2017**, *376*, 292.
71. Bourgeois-Vionnet, J.; Joubert, B.; Bernard, E.; Sia, M.A.; Pante, V.; Fabien, N.; Honnorat, J.; Streichenberger, N. Nivolumab-induced myositis: A case report and a literature review. *J. Neurol. Sci.* **2018**, *387*, 51–53.
72. Fox, E.; Dabrow, M.; Ochsner, G. A Case of Nivolumab-Induced Myositis. *Oncologist* **2016**, *21*, e3.
73. Saini, L.; Chua, N. Severe inflammatory myositis in a patient receiving concurrent nivolumab and azacitidine. *Leuk. Lymphoma* **2017**, *58*, 2011–2013.
74. Badovinac, S.; Korsic, M.; Zarković, K.; Muršić, D.; Roglic, M.; Jakopovic, M.; Samarzija, M. Nivolumab-induced synchronous occurrence of myositis and hypothyroidism in a patient with squamous cell lung cancer. *Immunotherapy* **2018**, *10*, 427–431.
75. Pushkarevskaya, A., Neuberger, U.; Dimitrakopoulou-Strauss, A.; Enk, A.; Hassel, J.C. Severe Ocular Myositis After Ipilimumab Treatment for Melanoma: A Report of 2 Cases. *J. Immunother.* **2017**, *40*, 282–285.
76. Lecouflet, M., Verschoore, M.; Giard, C.; Gohier, P.; Le Corre, Y.; Milea, D.; Martin, L. Orbital myositis associated with ipilimumab. *Ann. Dermatol. Venereol.* **2013**, *140*, 448–451.
77. Sheik Ali, S.; Goddard, A.L.; Luke, J.J.; Donahue, H.; Todd, D.J.; Werchniak, A.; Vleugels, R.A. Drug-associated dermatomyositis following ipilimumab therapy: A novel immune-mediated adverse event associated with cytotoxic T-lymphocyte antigen 4 blockade. *JAMA Dermatol.* **2015**, *151*, 195–199.
78. Yoshioka, M.; Kambe, N.; Yamamoto, Y.; Suehiro, K.; Matsue, H. Case of respiratory discomfort due to myositis after administration of nivolumab. *J. Dermatol.* **2015**, *42*, 1008–1009.
79. Vallet, H.; Gaillet, A.; Weiss, N.; Vanhaecke, C.; Saheb, S.; Touitou, V.; Franck, N.; Kramkimel, N.; Borden, A.; Touat, M.; et al. Pembrolizumab-induced necrotic myositis in a patient with metastatic melanoma. *Ann. Oncol.* **2016**, *27*, 1352–1353.
80. Haddox, C.L.; Shenoy, N.; Shah, K.K.; Kao, J.C.; Jain, S.; Halfdanarson, T.R.; Wijdicks, E.F.; Goetz, M.P. Pembrolizumab induced bulbar myopathy and respiratory failure with necrotizing myositis of the diaphragm. *Ann. Oncol.* **2017**, *28*, 673–675.
81. Bilen, M.A.; Subudhi, S.K.; Gao, J.; Tannir, N.M.; Tu, S.-M.; Sharma, P. Acute rhabdomyolysis with severe polymyositis following ipilimumab-nivolumab treatment in a cancer patient with elevated anti-striated muscle antibody. *J. Immunother. Cancer* **2016**, *4*, 36.
82. Gandiga, P.C.; Wang, A.R.; Gonzalez-Rivera, T.; Sreih, A.G. Pembrolizumab-associated inflammatory myopathy. *Rheumatology (Oxford)* **2018**, *57*, 397–398.
83. Diamantopoulos, P.T.; Tsatsou, K.; Benopoulou, O.; Anastasopoulou, A.; Gogas, H. Inflammatory Myopathy and Axonal Neuropathy in a Patient With Melanoma Following Pembrolizumab Treatment. *J. Immunother.* **2017**, *40*, 221–223.
84. Min, L.; Hodi, F.S. Anti-PD1 following ipilimumab for mucosal melanoma: Durable tumor response associated with severe hypothyroidism and rhabdomyolysis. *Cancer Immunol. Res.* **2014**, *2*, 15–18.

85. Carrera, W.; Baartman, B.J.; Kosmorsky, G. A Case Report of Drug-Induced Myopathy Involving Extraocular Muscles after Combination Therapy with Tremelimumab and Durvalumab for Non-Small Cell Lung Cancer. *Neuroophthalmology* **2017**, *41*, 140–143.
86. Kadota, H.; Gono, T.; Shirai, Y.; Okazaki, Y.; Takeno, M.; Kuwana, M. Immune Checkpoint Inhibitor-Induced Myositis: A Case Report and Literature Review. *Curr. Rheumatol. Rep.* **2019**, *21*, 10.
87. Ogawa, T.; Ishitsuka, Y.; Koguchi-Yoshioka, H.; Tanaka, R.; Fujisawa, Y.; Ishii, A.; Tamaoka, A.; Fujimoto, M. Polymyositis induced by PD-1 blockade in a patient in hepatitis B remission. *J. Neurol. Sci.* **2017**, *381*, 22–24.
88. Sekiguchi, K.; Hashimoto, R.; Noda, Y.; Tachibana, H.; Otsuka, Y.; Chihara, N.; Shiraishi, Y.; Inoue, T.; Ueda, T. Diaphragm involvement in immune checkpoint inhibitor-related myositis. *Muscle Nerve* **2019**, *60*.
89. Ong, S.; Chapman, J.; Young, G.; Mansy, T. Guillain-Barré-like syndrome during pembrolizumab treatment. *Muscle Nerve* **2018**, *58*, E8–E10.
90. De Maleissye, M.-F.; Saiag, P.; Nicolas, G. Pembrolizumab-Induced Demyelinating Polyradiculoneuropathy. *N. Engl. J. Med.* **2016**, *375*, 296–297.
91. Schneiderbauer, R.; Schneiderbauer, M.; Wick, W.; Enk, A.; Haenssle, H.; Hassel, J. PD-1 Antibody-induced Guillain-Barré Syndrome in a Patient with Metastatic Melanoma. *Acta Derm. Venereol.* **2017**, *97*, 395–396.
92. Fukumoto, Y., Kuwahara, M.; Kawai, S.; Nakahama, K.; Kusunoki, S. Acute demyelinating polyneuropathy induced by nivolumab. *J. Neurol. Neurosurg. Psychiatry* **2018**, *89*, 435–437.
93. Supakornnumporn, S.; Katirji, B. Guillain-Barre Syndrome Triggered by Immune Checkpoint Inhibitors: A Case Report and Literature Review. *J. Clin. Neuromuscul. Dis.* **2017**, *19*, 80–83.
94. Yost, M.D.; Chou, C.Z.; Botha, H.; Block, M.S.; Liewluck, T. Facial diplegia after pembrolizumab treatment. *Muscle Nerve* **2017**, *56*, E20–E21.
95. Jacob, A.; Unnikrishnan, D.C.; Mathew, A.; Thyagarajan, B.; Patel, S. A case of fatal Guillain–Barre syndrome from anti-PD1 monoclonal antibody use. *J. Cancer Res. Clin. Oncol.* **2016**, *142*, 1869–1870.
96. Wilgenhof, S.; Neyns, B. Anti-CTLA-4 antibody-induced Guillain-Barre syndrome in a melanoma patient. *Ann. Oncol.* **2011**, *22*, 991–993.
97. Kelly Wu, W.; Broman, K.K.; Brownie, E.R.; Kauffmann, R.M. Ipilimumab-induced Guillain-Barré Syndrome Presenting as Dysautonomia: An Unusual Presentation of a Rare Complication of Immunotherapy. *J. Immunother.* **2017**, 40, 196–199.
98. Gu, Y.; Menzies, A.M.; Long, G.V.; Fernando, S.; Herkes, G. Immune mediated neuropathy following checkpoint immunotherapy. *J. Clin. Neurosci.* **2017**, *45*, 14–17.
99. Nukui, T.; Nakayama, Y.; Yamamoto, M.; Taguchi, Y.; Dougu, N.; Konishi, H.; Hayashi, T.; Nakatsuji, Y. Nivolumab-induced acute demyelinating polyradiculoneuropathy mimicking Guillain-Barré syndrome. *J. Neurol. Sci.* **2018**, *390*, 115–116.
100. Thaipisuttikul, I.; Chapman, P.; Avila, E.K. Peripheral neuropathy associated with ipilimumab: A report of 2 cases. *J. Immunother.* **2015**, *38*, 77–79.
101. Tanaka, R.; Maruyama, H.; Tomidokoro, Y.; Yanagiha, K.; Hirabayashi, T.; Ishii, A.; Okune, M.; Inoue, S.; Sekine, I.; Tamaoka, A.; et al. Nivolumab-induced chronic inflammatory demyelinating polyradiculoneuropathy mimicking rapid-onset Guillain–Barré syndrome: A case report. *Jpn. J. Clin. Oncol.* **2016**, *46*, 875–878.
102. Sepúlveda, M.; Martinez-Hernandez, E.; Gaba, L.; Victoria, I.; Sola-Valls, N.; Falgàs, N.; Casanova-Molla, J.; Graus, F.; Martinez-Hernandez, E.; Sola-Valls, N.; et al. Motor polyradiculopathy during pembrolizumab treatment of metastatic melanoma. *Muscle Nerve* **2017**, *56*, E162–E167.
103. Aya, F.; Ruiz-Esquide, V.; Viladot, M.; Font, C.; Prieto-González, S.; Prat, A.; Arance, A. Vasculitic neuropathy induced by pembrolizumab. *Ann. Oncol.* **2017**, *28*, 433–434.
104. Manam, R.; Martin, J.L.; Gross, J.A.; Chaudhary, D.; Chowdhary, S.; Espinosa, P.S.; Santos, E.S. Case Reports of Pembrolizumab-induced Acute Inflammatory Demyelinating Polyneuropathy. *Cureus* **2018**, *10*, e3371.
105. Simsek, M.; Bilici, M.; Tekin, S.B. Creatinine kinase elevation and peripheral neuropathy during nivolumab treatment of a patient with metastatic renal cell carcinoma. *Indian J. Cancer* **2018**, *55*, 413.
106. Sakai, K.; Mochizuki, H.; Mochida, K.; Shiomi, K.; Amano, M.; Nakazato, M. A Case of Nivolumab-Induced Severe Mononeuropathy Multiplex and Rhabdomyolysis. *Case Rep. Med.* **2017**, *2017*, 1–4.

107. Zecchini, J.M., Kim, S.; Yum, K.; Friedlander, P. Development of Bell's Palsy After Treatment With Ipilimumab and Nivolumab for Metastatic Melanoma: A Case Report. *J. Immunother.* **2018**, *41*, 39–41.
108. Ziemann, D.; Frankel, A.E. Autoimmune Bell's Palsy Following Immunotherapy For Metastatic Melanoma:A Report of 2 Cases. *J. Immunother*. **2019**, *42*, 318–320.
109. Jinnur, P.; Lim, K.G. Severe Acute Orthopnea: Ipilimumab-Induced Bilateral Phrenic Nerve Neuropathy. *Lung* **2015**, *193*, 611–613.
110. Ghosn, J.; Vicino, A.; Michielin, O.; Coukos, G.; Kuntzer, T.; Obeid, M. A severe case of neuro-Sjögren's syndrome induced by pembrolizumab. *J. Immunother. Cancer* **2018**, *6*, 110.
